# LncRNA PCAT1 activates SOX2 and suppresses radioimmune responses via regulating cGAS/STING signalling in non‐small cell lung cancer

**DOI:** 10.1002/ctm2.792

**Published:** 2022-04-12

**Authors:** Yanping Gao, Nannan Zhang, Zihang Zeng, Qiuji Wu, Xueping Jiang, Shuying Li, Wenjie Sun, Jianguo Zhang, Yangyi Li, Jiali Li, Fajian He, Zhengrong Huang, Jinfang Zhang, Yan Gong, Conghua Xie

**Affiliations:** ^1^ Department of Radiation and Medical Oncology Zhongnan Hospital of Wuhan University Wuhan China; ^2^ Department of Biological Repositories Zhongnan Hospital of Wuhan University Wuhan China; ^3^ Tumor Precision Diagnosis and Treatment Technology and Translational Medicine Hubei Engineering Research Center Zhongnan Hospital of Wuhan University Wuhan China; ^4^ Frontier Science Center for Immunology and Metabolism Medical Research Institute School of Medicine Wuhan University Wuhan China; ^5^ Hubei Key Laboratory of Tumor Biological Behaviors Zhongnan Hospital of Wuhan University Wuhan China; ^6^ Hubei Cancer Clinical Study Center Zhongnan Hospital of Wuhan University Wuhan China; ^7^ Wuhan Research Center for Infectious Diseases and Cancer Chinese Academy of Medical Sciences Wuhan China

**Keywords:** cGAS/STING, NSCLC, PCAT1, radioimmune responses, SOX2

## Abstract

**Background:**

The expression of long non‐coding RNA (lncRNA) prostate cancer‐associated ncRNA transcripts 1 (PCAT1) is increased in non‐small cell lung cancer (NSCLC). It stimulates tumour growth and metastasis, but its role in the radioimmune responses remain unknown. We aimed to explore the impacts of PCAT1 on tumorigenesis and radioimmune responses and the underlying molecular mechanisms in NSCLC.

**Methods:**

Comprehensive bioinformatics analysis was performed to identify immunosuppressive lncRNAs involved with tumour invasion in NSCLC. The expression levels of PCAT1 were analysed by in situ hybridisation in 55 paired NSCLC tissues and adjacent normal tissues. Both loss‐ and gain‐of‐function assays were performed to examine the effects of PCAT1 and SOX2 on NSCLC cell behaviours in vivo and in vitro. Bioinformatic analyses, chromatin isolation by RNA purification (ChIRP) and dual‐luciferase reporter assays were applied to validate the regulatory effects of PCAT1 on SOX2 expression. Chromatin immunoprecipitation, luciferase and rescue assays were utilised to identify the relationship between SOX2 and the cGAS/stimulator of interferon genes (STING) signalling.

**Results:**

PCAT1 was immunosuppressive and related with NSCLC invasion. Increased PCAT1 was negatively correlated with immune cell infiltration in NSCLC. PCAT1 knockdown restrained proliferation, increased apoptosis, and repressed cell metastasis in vivo and in vitro. PCAT1 activated SOX2 that accelerated tumorigenesis and immunosuppression. SOX2 promoted tumour growth through inhibiting cytotoxic T‐cell immunity. Moreover, SOX2 restrained cGAS transcription and hampered downstream type I interferon (IFN)‐induced immune responses. Inhibition of PCAT1/SOX2 in collaboration with radiation further inhibited tumour growth, and initiated the cGAS/STING signalling pathway, which enhanced the immune responses of radiotherapy in NSCLC.

**Conclusions:**

PCAT1/SOX2 axis promoted tumorigenesis and immunosuppression through inhibition of cGAS/STING signalling‐mediated T‐cell activation. Inhibition of PCAT1 and SOX2 synergised with radiotherapy to activate the immune response and could serve as potential therapeutic targets.

## Graphical abstract

- LncRNA PCAT1 is highly expressed immunosuppressive in NSCLC via downregulating cGAS/STING signalling pathway.
- LncRNA PCAT1 upregulates SOX2 transcription and SOX2 downregulates cGAS transcription.
- LncRNA PCAT1 suppresses radioimmune responses through SOX2/cGAS/STING pathway and its downregulation increases the inhibitory effects of radiotherapy in NSCLC.

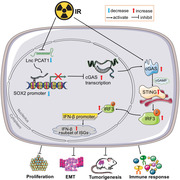

## 1 INTRODUCTION

Lung cancer is still the leading cause of cancer patient death.^1^ Non‐small cell lung cancer (NSCLC), accounting for approximately 80% cases, is the most common subtype of lung cancer.^2^ The 5‐year relative survival of NSCLC patients remains pitiable, with only 21% for all stages combined.^2^ Although early‐stage patients could benefit from radical surgery and radiotherapy, and had favourable prognosis, the majority of patients diagnosed with locally advanced or metastatic diseases had dismal prognosis.^3^ The development of targeted therapy and innovative immunotherapy has evolutionally reshaped the landscape of NSCLC treatment. In particular, immune checkpoint treatment represented by anti‐PD‐1/PD‐L1 antibodies greatly improved overall survival (OS) and yielded long‐term diseases control in NSCLC. However, the overall response rate of immunotherapy remains low. Only approximately 20% patients could potentially benefit from immune checkpoint treatment. Identifying novel predictive biomarkers and amplifying immunotherapeutic responses rate has become crucial for this deadly cancer.

Recent studies and clinical investigations indicated that radiotherapy not only induced DNA damage and cell death that contributed to local tumour regression, but also promoted an immune‐stimulatory microenvironment and systemic immune responses that resulted in distant tumour control.^4^ It has been recommended that the therapeutic efficacy of ionising radiation (IR) may be optimised via engaging the innate immune system.^5^ Radiotherapy was reported to enhance anti‐tumour responses of immunotherapy via inducing DNA damage and modulating tumour microenvironment (TME).9.10 Ionising radiation (IR) augmented antigen processing,^6^ mounted sensibility of tumour cells to cytotoxic T‐cell killing,^7^ and stimulated tumour cells to secret immunogenic molecules.^8^ Cyclic GMP‐AMP (cGAMP) synthase (cGAS) and stimulator of interferon genes (STING) bridge IR‐induced DNA damage with CD8+ cytotoxic T cell‐mediated radioimmune responses. The activation of cGAS/STING axis induces innate immune factors including type I interferons (IFNs) and other cytokines.^11^ More comprehensive knowledge on the regulation of cGAS/STING signalling can availably harness anti‐tumour immunity following IR. Accordingly, more extensive characterisation of immune responses and exploration on the underlying mechanism are required to enhance the combination of radiotherapy and immunotherapy in NSCLC.

Recently, increasing researches indicated that long non‐coding RNAs (lncRNAs) could be vital players in immune cell development and immune responses, and also influence the TME.^12^, ^13^ Meanwhile, the lncRNAs may be unique markers of tumour prognosis and provide potential targets for cancer immunotherapies.^14^, ^15^ LncRNA prostate cancer‐associated ncRNA transcripts 1 (PCAT1) was originally described as a prostate‐specific regulator of cell proliferation in prostate cancer in 2011.^16^ Subsequent researches showed that PCAT1 was deregulated in numerous human cancers and associated with carcinogenesis, clinicopathological features and prognosis.^17^, ^18^, ^19^ Recently, PCAT1 was testified to be upregulated in NSCLC and promoted cell proliferation and metastasis.^20^, ^21^ However, the roles of PCAT1 in innate immune responses have not been reported in NSCLC. The sex‐determining region Y‐related high‐mobility group box 2 (SOX2) is one of PCAT1 target genes. SOX2 was reported to regulate T cells and tumour‐associated neutrophils, as well as fine‐tunes cancer immunity.^22^, ^23^ In addition, SOX2 dampened anti‐tumour immunity through inhibition of stimulator of interferon genes (STING) signalling, which is critical for the induction of type I IFN and stimulation of both innate and adaptive immunity.^24^ However, the role of SOX2 in NSCLC development and immune response remains unclear.

Using various cohorts of database of NSCLC, we identified and validated PCAT1 as an immunosuppressive and invasion‐related lncRNA in NSCLC. Increased PCAT1 was negatively correlated with immune cell infiltration, and responsible for the tumorigenesis and progression of NSCLC. Further studies focussed on its functions and potential mechanisms in the carcinogenesis and cancer immunity of NSCLC. PCAT1 suppressed radioimmune responses via regulating the cGAS/STING signalling pathway in NSCLC cells, and SOX2 was involved in this process. PCAT1 deficiency inhibited NSCLC growth and tumorigenicity in vivo, and SOX2 overexpression dampened IR‐induced adaptive immune responses and promoted tumour growth. Our results suggested that PCAT1 dictated NSCLC development and radioimmune responses via regulating SOX2/cGAS/STING axis.

## 2 MATERIALS AND METHODS

### 2.1 Bioinformatic analysis

A total of 995 patients from cohorts for lung adenocarcinoma (LUAD) and lung squamous cell carcinoma (LUSC) with complete RNA sequencing (RNA‐seq) and OS were recruited with annotations from The Cancer Genome Atlas (TCGA). The RNA‐seq was normalised by TPM & log_2_ (*x*+1) and downloaded by Xena database.^25^ The Kaplan‐Meier survival curve analysis was constructed, and the log‐rank test was applied to contrast different OS between the high‐ and low‐expression groups. The Gene Set Enrichment Analysis (GSEA) was downloaded from molecular signatures database. GSEA was executed by R “clusterProfiler” packages based on GO corpus.^26^ The significant level of false discovery rate (FDR) was 0.05. The multivariate Cox regression analysis was conducted to validate PCAT1 as an independent prognostic factor. R survival package was used to perform Cox proportional hazards regression. Immune cell infiltration was estimated by MCPcounter.^27^ The sample size was determined as the power (1‐β) ≥ .8.^28^

### 2.2 Tissue chip

The LUAD tissue chip was attained from Shanghai Outdo Biotech (Shanghai, China), which included 55 carcinomas and paired adjacent tissues. All patients were pathologically diagnosed as LAUD.

### 2.3 In situ hybridisation

In situ hybridisation (ISH) was used to examine the expression and location of PCAT1 in NSCLC tissues. Paraffin slides were dewaxed in paraformaldehyde and washed in an ethanol gradient. Triton‐X100 (0.5%) and proteinase K were used to expose the nucleic acids before hybridisation. Next, PCAT1 probes were used to incubate the slides at 42°C for 16 h. After 30 min blocking solution, the specimens were incubated with antibodies at constant 4 °C overnight. The hematoxylin c solution was then used to stain the specimens. Quantification was measured as the product of density and area. According to the maximum effect, the criteria to make the cutoff for the two groups were the threshold with the largest difference.

### 2.4 Cells

Human bronchial epithelial cells (BEAS‐2B), five NSCLC cell lines including A549, PC9, H1975, H520, H1299 and Lewis lung carcinoma (LLC) cells were used for experiment, which were cultured in RPMI‐1640 or DMEM (Gibco, Grand Island, NY, USA) added with 10% fetal bovine serum (HyClone, USA) at 37°C and 5% CO_2_. All of these cells were purchased from the Type Culture Collection of the Chinese Academy of Sciences (Shanghai, China).

### 2.5 Plasmids and lentiviruses

Small interfering RNAs (siRNAs) targeting *PCAT1* and *SOX2* were synthesised by Shanghai Gene pharm Technologies (GenePharma, Shanghai, China). The corresponding siRNA sequences are shown in Table S1. For gene overexpression, full length human *PCAT1* was cloned into the pcDNA3.1 vector (Invitrogen, Shanghai, China). *SOX2* was cloned into the pEX‐4 expression vector (GenePharma). The cGAS overexpression plasmid was gifted from Dr. Xiaolian Cai. For in vivo studies, *PCAT1* depletion, *SOX2* depletion and *SOX2* overexpression cells were generated with lentiviruses. Short hairpin (sh) RNA lentivirus targeting *PCAT1* (LV‐sh‐PCAT1) and *SOX2* (LV‐sh‐SOX2) as well as *SOX2* overexpression lentiviruses (LV‐SOX2) were purchased from GENECHEM (Shanghai, China).

### 2.6 RNA sequencing

Ribo‐Zero Magnetic Kit (Plant Leaf, Madison,Wisconsin, USA) was applied to treat total RNA for depleting rRNA. The First Strand Master Mix (Invitrogen) was used to fragment the retrieved RNA. After library construction, Ampure XP Beads were used to purify the polymerase chain reaction (PCR) products. The average molecule length was determined using the Agilent 2100 bioanalyzer instrument, and the libraries were quantified by quantitative real‐time polymerase chain reaction (qRT‐PCR) and sequenced pair end on the Illumina sequencing system.

### 2.7 RNA extraction and qRT‐PCR

SuperScript II (Vazyme, Nanjing, China) was used to synthesise cDNA according to the manufacturer's instructions. qRT‐PCR was performed using the ChamQTM SYBR qPCR Master Mix (Vazyme, Nanjing, China). The primers are presented in Table S2.

### 2.8 Immunofluorescence

Cells seeded on coverslips were fixed in 4% formaldehyde at room temperature for 15 min. They were then permeabilised in .1% Triton X‐100 and blocked in 5% bovine serum albumin at room temperature for 1 h. Cells were incubated with primary antibodies (Table S3) at 4 °C overnight, and then incubated with appropriate secondary antibodies. Cell nuclei were visualised with DAPI (0.5 μg/ml) in the dark for 15 min. Images were obtained under a fluorescence microscope (MSHOT, China).

### 2.9 Immunoblotting

Cells were lysed by RIPA buffer (Beyotime, Shanghai, China) with phosphatase inhibitor (Sigma‐Aldrich, St. Louis, MO, USA) on ice for 30 min. We added equal amounts of proteins into the 10% SDS‐polyacrylamide gels and polyvinylidene fluoride membranes were used to transfer. After 2 h blocking using 5% non‐fat milk, the transferred membranes were incubated with primary antibodies at 4°C for 12–16 h. The membranes were then incubated with secondary antibodies after washing five times with TBST for 1.5 h. In a gel imaging analyser, we detected the antibody‐bound proteins with enhanced chemiluminescence detection kit (Bio‐Rad, Hercules, CA, USA). The antibodies are presented in Table S3.

### 2.10 Chromatin isolation by RNA purification

The cells were carefully harvested and cross‐linked with 1% glutaraldehyde followed by sonication. The lysates were subjected to specifically pulldown with streptavidin beads, and the bound DNA was quantified with RT‐qPCR with SOX2 promoter‐specific primers (Table S4). The Magna ChIRP Chromatin Isolation by RNA Purification Kit (Millipore, USA) was used to determine the interaction between PCAT1 and the promoter of SOX2 according to the manufacturer's instructions.^29^, ^30^

### 2.11 Dual‐luciferase reporter assay

The PGL3B firefly luciferase vectors comprised empty group, wild‐type *SOX2* and mutant *SOX2*, both co‐transfected with PCAT1 plasmids. After transfection for 48 h, luciferase activity assays for PCAT1 target validation were performed according to the manufacturer's instructions (Promega). The psiCHECK2 dual‐luciferase SOX2 expression vector was used to assess the putative regulation of SOX2 on the target sites. The luciferase activities were evaluated using a dual‐luciferase reporter 48 h after transduction.

### 2.12 Chromatin immunoprecipitation

NSCLC cells at 60%–80% confluency in 10‐cm petri dishes were collected. After cross‐linking with 1% formaldehyde at room temperature, the cells were resuspended in lysis buffer including protease inhibitor on ice for 30 minutes. The lysates were sonicated and incubated with anti‐SOX2 antibodies overnight. Chromatin immunoprecipitation (ChIP) was performed using the ChIP Kit (Thermo) according to the manufacturer's protocol. Protein G agarose was used to capture the lysates. After eluted from the beads, the chromatin DNA was treated with proteinase K and extracted using phenol‐chloroform. Real‐time PCR was implemented to amplify the target sequences from the immunoprecipitated DNA samples and the input with specific primers (cGAS‐F, GGGCAAGAAACACGCTCCAGTC; cGAS‐R, GCTCCATTTGCTTGTGGGTGATTG).

### 2.13 Animals

BALB/c nude (5‐6 weeks) and C57BL/6 mice (5‐6 weeks) were purchased from the Vital River Laboratories Animal Technology (Beijing, China). PC9‐LV‐NC or PC9‐LV‐sh‐PCAT1 cells (3 × 10^6^ / 100 μl) were collected and subcutaneously injected into the right flanks of BALB/c nude mice. In the irradiation experiments, stably transfected LLC cells (1 × 10^6^ / 100 μl) were subcutaneously injected into the right flank of C57BL/6 mice. The tumour volume was measured every other day. When tumours reached approximately 200 mm^3^, mice were irradiated by the small animal radiation research platform (PXI X‐RAD 225Cx, Gulmay, CT, USA). To make the radiation zone and dose consistent between tumours, outliers with more than three times larger or smaller than the mean volume of the tumours were excluded. When the tumours reached 2000 mm^3^ or necrotised, mice were euthanised. After 5 weeks, the mice were anesthetised and bioluminescence images were taken with IVIS Lumina XRMS Series III (PerkinElmer, USA). Animal operations were approved by Institutional Animal Care and Use Committee of Wuhan University.

### 2.14 Flow cytometry

Cells were washed with PBS and resuspended in binding buffer (1 × 10^6^ cells / ml). For apoptosis, 100 μl cell suspension was stained with fluorescein isothiocyanate‐Annexin V for 20 min and propidium iodide (PI) for 5 min (BD, Franklin Lake, NY, USA). For cell cycle, cells were treated with the cell cycle staining kit (MultiSciences, China), and then analysed with Flow cytometry (Beckman). For immunophenotyping of TME, tumour tissues (*n* ≥ 6) were minced mechanically and digested with collagenase IV (Sigma‐Aldrich) to obtain single cell suspensions, from which the absolute live cell count was determined by incubating in Fixable Viability Stain 700. Cells were stained with fluorescein‐conjugated antibodies (Table S3) at 4 °C for 30 min and analysed using FACS Aria III flow cytometry (BD). Data were analysed with FlowJo software.

### 2.15 Statistical analyses

Data were presented as mean ± SEM from at least three independent repeats. The Mann‐Whitney U test was used to compare continuous data between groups. The Student's *t*‐test was used to compare two variables and numerical values. The one‐way ANOVA was used to analyse multiple group comparisons of continuous data. *p* < .05 was considered as statistical difference.

## 3 RESULTS

### 3.1 Increased PCAT1 was negatively correlated with immune infiltration in NSCLC

**FIGURE 1:**
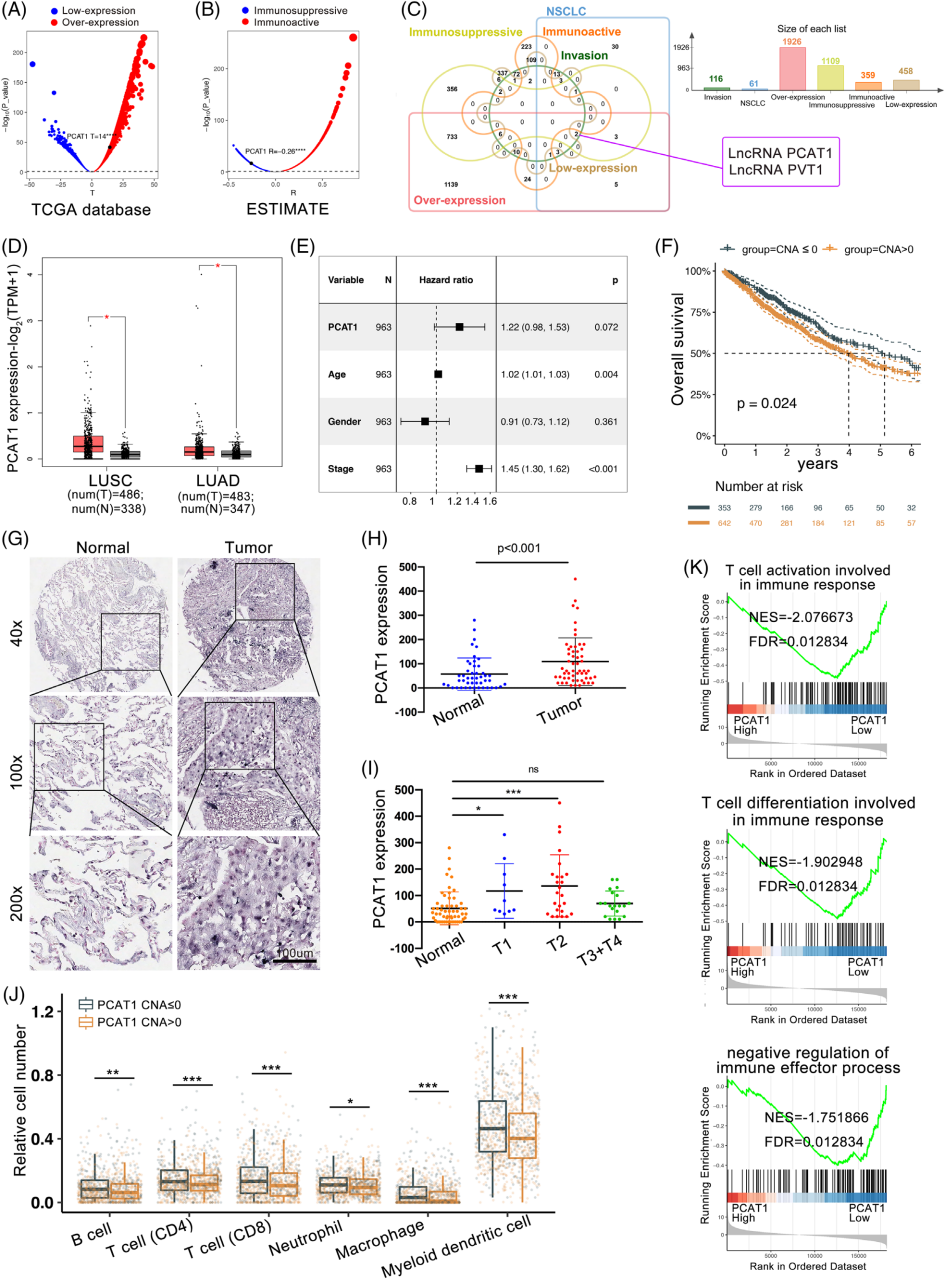
Increased prostate cancer‐associated ncRNA transcripts 1 (PCAT1) was negatively correlated with immune cell infiltration in non‐small cell lung cancer (NSCLC). (A) PCAT1 was overexpressed in NSCLC tumour versus non‐tumour tissues (*p* < .001). (B) The immune scores of each NSCLC patient were calculated through ESTIMATE. PCAT1 was immunosuppressive. (C) Wenn plot of lncRNAs with different expression levels and immune functions in NSCLC. Only PCAT1 and PVT1 were immunosuppressive and involved in NSCLC invasion. (D) The expression levels of PCAT1 were significantly higher in both lung squamous cell carcinoma (LUSC) and lung adenocarcinoma (LUAD) tissues compared with non‐tumour tissues. (E) PCAT1 was an independent prognostic element with multivariate cox regression model. (F) The Kaplan‐Meier survival curve analysis for NSCLC patients based on PCAT1 copy number alteration (CNA). (G) In situ hybridisation (ISH) was performed in 55 pairs of paraffin‐embedded tissues. Expression of PCAT1 was significantly higher in NSCLC tissues compared to adjacent noncancerous tissues (*p* < .01). (H) Representative ISH images of normal and tumour tissues with PCAT1 staining. Scale bar, 200 μm. (I) The expression of PCAT1 in the T1 and T2 phases was significantly increased (*p* < .05) compared with the adjacent normal tissues. (J) The immune cell infiltration of NSCLC was estimated via MCPcounter algorithm. (K) Gene Set Enrichment Analysis (GSEA) for the enriched gene sets in the PCAT1 expression. NES, normalised enrichment score

Immune‐related lncRNAs regulate the immunosuppressive activity and function as possible oncogenes to promote cancer development. Yet, their roles in NSCLC are little known. To identify novel oncogenic lncRNAs that suppress immune essentials for NSCLC, bioinformatic data sets (TCGA‐LUAD, TCGA‐LUSC) were applied to identify differentially expressed lncRNAs between NSCLC and the corresponding non‐tumour samples. First, we performed *t*‐test of tumour versus non‐tumour RNAs for the 25 598 lncRNAs, of which the 1926 lncRNAs was upregulated in the NSCLC (*p* < .001, Figure 1A). Subsequently, we calculated the immune scores of each NSCLC patient through ESTIMATE, and 1109 lncRNAs negatively correlated with immune scores were identified through Pearson's correlation (R < ‐.2, *p* < .001, Figure 1B). Next, we attained 61 NSCLC‐related lncRNAs and 116 invasion‐related lncRNAs from lncSEA database. PCAT1 and PVT1 were immunosuppressive and invasion‐related in NSCLC (Figure 1C). While the roles of PVT1 were well documented, the molecular mechanisms of PCAT1 in immune repression have not been reported in NSCLC. To identify the roles of PCAT1 in NSCLC, we analysed its expression levels in NSCLC tissues. The transcriptome sequencing data (*n* = 594) from both The Cancer Genome Atlas (TCGA) LUAD and TCGA LUSC cohorts showed increased levels of *PCAT1* in NSCLC tissues (Figure 1D). Multivariate cox regression indicated that the copy number alteration (CAN) of *PCAT1* was a prognostic factor independent of age, gender and cancer stage (Figure 1E). The CNA of *PCAT1* was linked to unfavourable OS in NSCLC patients (Figure 1F). Further analysis indicated that higher PCAT1 levels were correlated with increased CNA (Figure S1A), suggesting the relationship between PCAT1 expression and NSCLC prognosis. The expression of PCAT1 in 55 paired NSCLC and adjacent non‐tumuor tissues was detected by ISH. A noteworthy upregulation of PCAT1 was observed in NSCLC (Figure 1G,H and Table S5). According to the one‐way ANOVA, the expression level of PCAT1 in the T1 and T2 phases was significantly increased (*p* < .05) compared with the adjacent group (Figure 1I). PCAT1 was slightly upregulated in the T3+T4 phases, and the insignificance might be affected by the complex covariates of clinical data. According to the chi‐square test, there is no significant correlation between PCAT1 expression and each index (Table S6). To explore the potential TME changes caused by PCAT1 amplification, we estimated the immune infiltrations of NSCLC using MCPcounter algorithm. The PCAT1 amplification was negatively correlated with immune infiltration, including CD4+ and CD8+ memory T cells, as well as macrophages (Figure 1J). The PCAT1 activity in immune pathways was calculated based on the modified Gene Set Enrichment Analysis (GSEA). PCAT1 was associated with T cell and immune signals of GSEA: T‐cell activation, IFN‐β T‐cell activation and negative regulation of humoural immune response (FDR < .05, Figure 1K). Taken together, these correlates suggested that serviceable roles of PCAT1 as oncogenes can exert immunosuppressive function in NSCLC.

### 3.2 PCAT1 depletion inhibited NSCLC cell growth, increased apoptosis and induced migration

The relative expression levels of PCAT1 were determined in various NSCLC cells and BEAS‐2B cells. A significantly higher expression of PCAT1 was detected in NSCLC cells (Figure S2A). PC9 and H1975 cell were selected to investigate the effects of PCAT1 depletion on NSCLC cell behaviours (Figure S2B). PCAT1 silencing inhibited NSCLC cell colony formation and proliferation (Figure S2C,D). Cell apoptosis and G1 arrest were promoted upon PCAT1 deficiency in PC9 and H1975 cells (Figure S2E,F). Moreover, PCAT1 depletion increased NSCLC cell migration and invasion (Figure S3A,B). Imuunofluorescence and immunoblotting indicated that PCAT1 downregulation led to increased E‐cadherin, and reduced N‐cadherin and vimentin in PC9 and H1975 cells (Figure S3C,D). These results suggested vital roles of PCAT1 in NSCLC cell behaviours.

### 3.3 PCAT1 silencing activated cGAS/STING signalling in NSCLC cells

**FIGURE 2:**
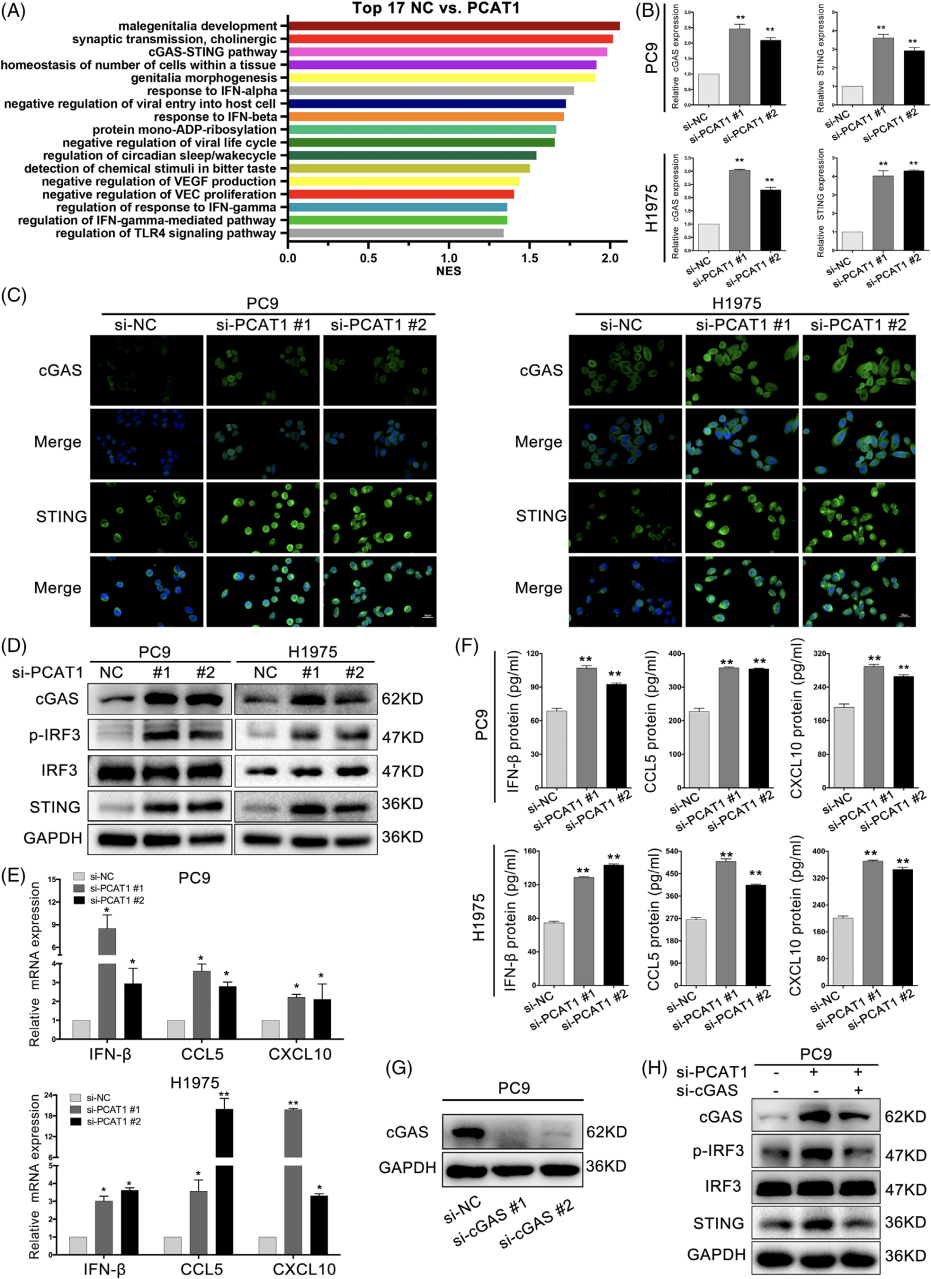
Prostate cancer‐associated ncRNA transcripts 1 (PCAT1) silencing induced cyclic GMP‐AMP synthase/stimulator of interferon genes (cGAS/STING) signalling in non‐small cell lung cancer (NSCLC) cells. (A) RNA sequencing (RNA‐seq) revealed high correlation between PCAT1 and cGAS/STING pathway. (B) PCAT1 depletion increased mRNA levels in PC9 and H1975 cells. (C) Representative immunofluorescence of cGAS and STING in PC9 and H1975 cells with PCAT1 deficiency. Scale bar, 50 μm. (D) Representative immunoblotting of cGAS, STING, p‐IRF3 and IRF3 in NSCLC cells with PCAT1 knockdown. (E) PCAT1 silencing decreased the mRNA levels of IFNβ, CCL5 and CXCL10 in PC9 and H1975 cells. (F) The secretion of IFNβ, CCL5 and CXCL10 was measured by enzyme‐linked immunosorbent assay (ELISA) in PCAT1‐deficient NSCLC cells. (G) Representative immunoblotting of cGAS in PC9 cells with cGAS knockdown. (H) cGAS depletion impaired the induction of cGAS/STING signalling pathway by PCAT1 downregulation. *n* = 3; *, *p* < .05; **, *p* < .01

Given that type I IFN signalling induces CD8+ T‐cell responses against tumours,^31^ and that cGAS/STING signalling pathway promotes type I IFN production and adaptive immunity,^32^ the expression of cGAS/STING, IFN‐b and downstream chemokines were detected in NSCLC cells. We overexpressed PCAT1 in PC9 cells (Figure S1B) and examined the related signalling pathways through RNA‐seq(Figure 2A). The cGAS/STING pathway was among the top three signalling pathways in GSEA, and the only one involved in IFN production and adaptive immunity among them. To further explore the underlying mechanisms, we surveyed the effects of PCAT1 downregulation on DNA damage. The DNA damage marker γ‐H2AX was significantly increased upon PCAT1 silencing. In addition, PCAT1‐deficient PC9 and H1975 cells had more DNA damage (Figure S4A). The percentages of tail DNA content were augmented notably in PCAT1‐deficient PC9 and H1975 cells (Figure S4B,C). Further studies confirmed that PCAT1 silencing boosted the accumulation of dsDNA in cytosol (Figure S4D). These results suggested that PCAT1 silencing provoked DNA damage in PC9 and H1975 cells. Enhanced expression of cGAS and STING were observed in PCAT1‐silencing NSCLC cells at both mRNA and protein levels (Figure 2B‐D and S6A‐E). The immunofluorescent images of STING are presented in Figure S5 to validate antibody staining. Accordingly, the phosphorylation of cGAS/STING signalling downstream protein IRF3 was also increased (Figure 2D and S6E). An upregulation of IFN‐β, CCL5 and CXCL10 by the inhibition of PCAT1 was also observed at both mRNA and protein levels (Figure 2E,F and S6F‐G). To confirm the regulation of cGAS/STING pathway by PCAT1, cGAS was depleted in the PCAT1‐deficient PC9 cells. Immunoblotting suggested that cGAS knockdown impaired the induction of cGAS/STING signalling induced by PCAT1 downregulation (Figure 2G,H). All the results indicated that PCAT1 downregulation induced cGAS/STING signalling pathway in NSCLC cells.

### 3.4 PCAT1 positively regulated SOX2 expression by direct induction of *SOX2* transcription

**FIGURE 3:**
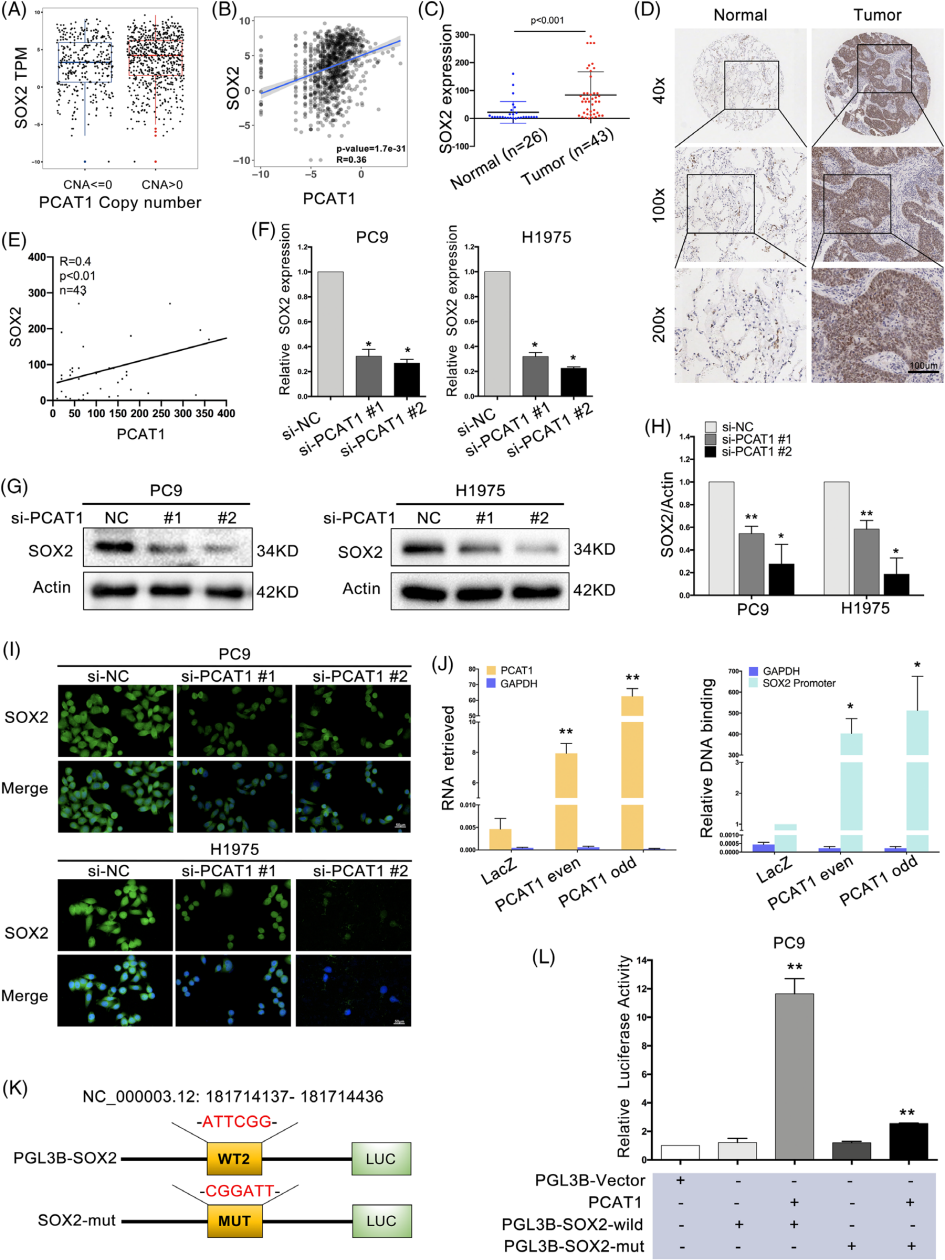
Prostate cancer‐associated ncRNA transcripts 1 (PCAT1) positively regulated sex‐determining region Y‐related high‐mobility group box 2 (SOX2) expression by direct induction of *SOX2* transcription. (A,B) The correlation analysis between PCAT1 and SOX2 in non‐small cell lung cancer (NSCLC) patients was performed with The Cancer Genome Atlas (TCGA) database. (C) SOX2 expression was significantly higher in NSCLC tissues compared to the adjacent normal tissues. (D) Representative immunohistochemistry (IHC) images of SOX2 in normal and NSCLC tissues. Scale bar, 200 μm. (E) PCAT1 is positively correlated with SOX2 in NSCLC tissues. (F‐H) PCAT1 silencing downregulated SOX2 mRNA and protein levels in NSCLC cells. (I) Representative immunofluorescence of SOX2 in PC9 and H1975 cells with PCAT1 depletion. Scale bar, 50 μm. (J) The results of chromatin isolation by RNA purification (ChIRP) showed that PCAT1 bound to SOX2 promoter. (K) Schematic representation of PCAT1 promoter with the putative SOX2‐binding sites and the sequences of the point mutations. (L) Luciferase assay demonstrated that PCAT1 positively regulated SOX2. *n* = 3; *, *p*< .05, **, *p* < .01

To investigate the molecular mechanism how PCAT1 regulated cGAS/STING signalling, we searched published literatures and performed bioinformatic analyses. Previous report suggested that PCAT1 promotes NSCLC stemness via upregulating SOX2.^33^ SOX2 was reported to regulate STING expression in head and neck squamous cell carcinoma.^24^ In TCGA NSCLC gene expression database, PCAT1 copy number and expression were positively correlated with SOX2 expression (Figure 3A,B). Compared with adjacent normal tissues, a significant rise of SOX2 was detected in NSCLC tissues (Figure 3C,D and Table S7). The correlation between SOX2 expression and clinicopathological characteristics was not significantly different with the chi‐square test (Table S8). In addition, PCAT1 expression was positively correlated with SOX2 expression in NSCLC tissues (Figure 3E). Inhibition of PCAT1 led to a decrease of SOX2 mRNA and protein levels (Figure 3F‐I). Previous studies showed that lncRNAs can serve to guide transcription factors to a specific sequence.^34^ We designed primers and performed ChIRP assay to explore whether PCAT1 possessed an ability to bind to SOX2 promoter sites. Biotin‐labelled PCAT1 could capture SOX2 promoter sequences (Figure 3J). To elucidate whether PCAT1 influenced the transcriptional activity of SOX2 via binding to SOX2 promoter, we generated an SOX2 promoter mutation‐containing pGL3B reporter vector and co‐transfected with PCAT1 ectopic expression into PC9 cells (Figure 3K). While co‐transfection of wild‐type SOX2 along with PCAT1 significantly increased luciferase activity, the stimulatory roles of PCAT1 on luciferase activity were attenuated when the binding sequences in the promoter area were mutated (Figure 3L). These results indicated that PCAT1 regulated SOX2 expression via binding to its promoter.

### 3.5 Knockdown of SOX2‐inhibited tumorigenesis in NSCLC

SOX2 was reported to be an oncogene that promoted the malignant behaviours of lung cancer cells.^35^, ^36^, ^37^, ^38^ In TCGA database, SOX2 mRNA levels were significantly exceeded in both LUAD and LUSC than those in normal samples (Figure S7A). Its expression was also upregulated in NSCLC cells (Figure S7B). To verify its roles in NSCLC cells, siRNAs and overexpression lentiviruses were used to down‐ and upregulated SOX2 in NSCLC cells (Figure S7C‐E). SOX2 knockdown significantly suppressed NSCLC cell viability and colony formation, and stimulated apoptosis (Figure S7F‐H). Moreover, loss of SOX2‐repressed NSCLC cell migration and invasion (Figure S8A,B). SOX2 silencing upregulated E‐cadherin and downregulated N‐cadherin and Vimentin (Figure S8C). Taken together, these observations suggested that SOX2 conspicuously stimulated NSCLC progression via enhancing cell proliferation, apoptosis, migration and invasion.

### 3.6 SOX2 inhibited cGAS/STING signalling in NSCLC

**FIGURE 4:**
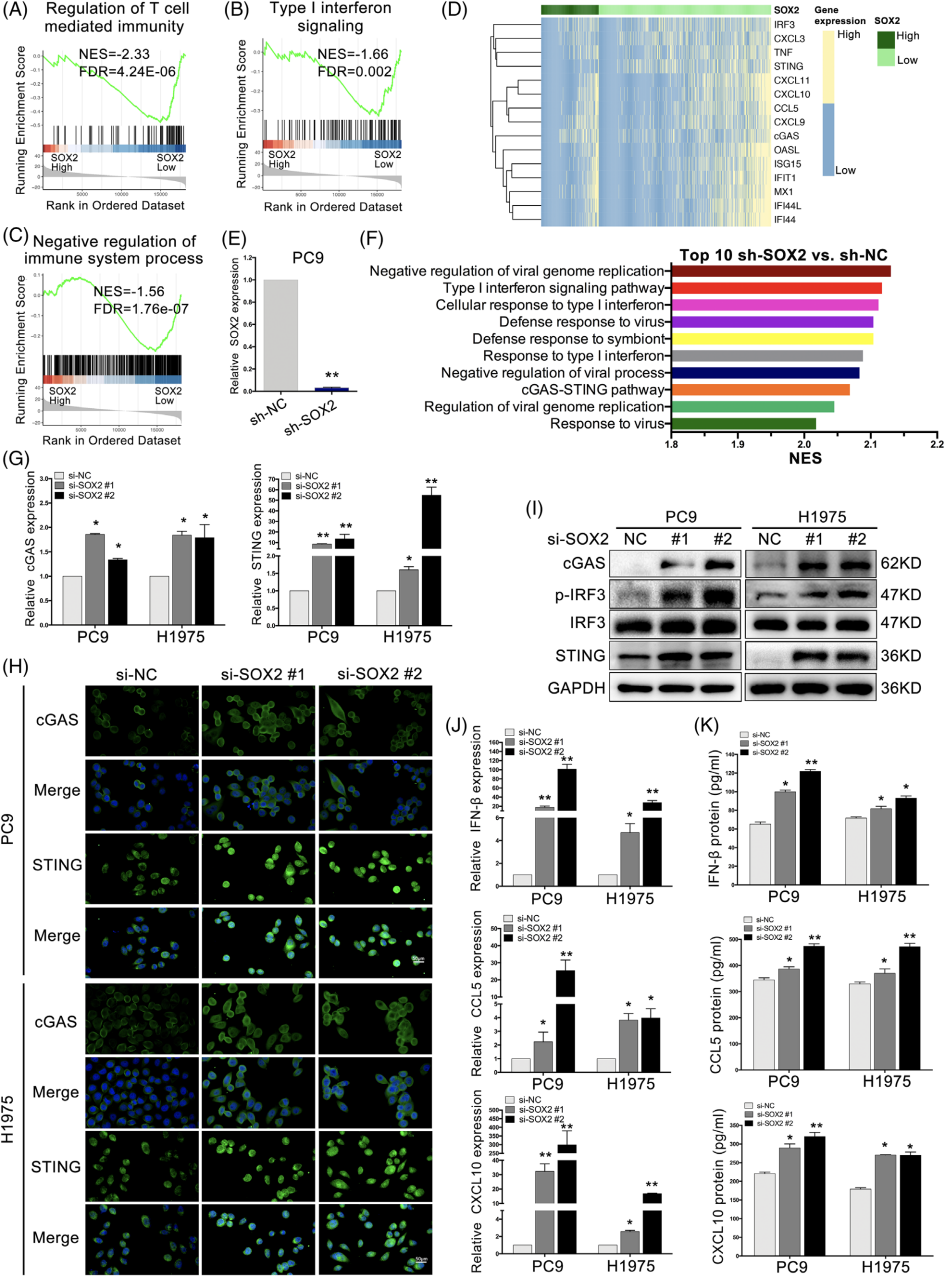
Sex‐determining region Y‐related high‐mobility group box 2 (SOX2) inhibited cGAS/STING signalling in non‐small cell lung cancer (NSCLC). (A‐C) The expressions of SOX2 linked to signals including T‐cell mediated immunity, type I interferon (IFN) signalling and negative regulation of immune system process (false discovery rate [FDR] < .05). (D) Heat map of cGAS/STING representative gene expression between the low and high SOX2 expression groups. (E) SOX2 was significantly downregulated by shRNAs in PC9 cells for RNA sequence (RNA‐seq) (F) RNA‐seq revealed the correlation between SOX2 silencing and cGAS/STING pathway. (G) The mRNA levels of cGAS and STING were detected by quantitative real‐time polymerase chain reaction (qRT‐PCR) after si‐SOX2 transfection in NSCLC cells. (H) Representative immunofluorescence of cGAS and STING in PC9 and H1975 cells transfected with si‐SOX2. Scale bar, 50 μm. (i) Representative immunoblotting of cGAS, STING, p‐IRF3 and IRF3 after SOX2 silencing. (J) The mRNA levels of IFNβ, CCL5 and CXCL10 were detected by qRT‐PCR in SOX2‐deficient cells. (K) The secretion of IFNβ, CCL5 and CXCL10 were assayed by enzyme‐linked immunosorbent assay (ELISA) in PCAT1‐deficient cells. *n* = 3; *, *p* < .05; **, *p* < .01

To investigate whether SOX2 potentially mediated PCAT1‐induced immune modulation in NSCLC, we explored the impacts of SOX2 via performing the GSEA in TCGA data set of NSCLC. The most significantly altered pathways were identified, including T cell‐mediated immunity, type I IFN signalling and downregulation of immune process (FDR < .05, Figure 4A‐C). SOX2 expression, PCAT1 expression and CNA were negatively correlated with major cellular markers of T cells, cytotoxic lymphocytes and B cells as identified by MCPcounter (Figure S9A). To well recognize how SOX2 potentiates immune escape and targets the IFN‐I pathway, we verified the expression of SOX2 and cGAS/STING pathway through R heatmap package from TCGA database. The cGAS/STING‐related genes and IFN‐stimulated genes (ISGs) were significantly repressed in the high SOX2 group (Figure 4D and S9B). Meanwhile, we downexpressed SOX2 in PC9 cells (Figure 4E) and examined the related signalling pathways through RNA‐seq (Figure 4F). The cGAS/STING pathway was among the top 10 signalling pathways in GSEA, and type I interferon signalling pathway also was affected. We next assessed the effects of SOX2 downregulation on DNA damage. The DNA damage marker γ‐H2AX was significantly increased upon SOX2 deficiency (Figure S10A). In addition, SOX2‐deficient PC9 and H1975 cells had more DNA damage (Figure S10B). The percentages of tail DNA content were increased notably (Figure S10C,D). Furthermore, SOX2 silencing increased dsDNA accumulation in cytosol (Figure S10E). SOX2 silencing activated cGAS/STING signalling and promoted the expression of IFN‐β, CCL5 and CXCL10 in NSCLC cells (Figure 4G,K and S11A‐G). To confirm the regulation of cGAS/STING pathway by SOX2, cGAS was depleted in the SOX2‐deficient PC9 cells. Immunoblotting suggested that cGAS knockdown impaired the induction of cGAS/STING signalling induced by SOX2 downregulation (Figure S11H). These results suggested that SOX2 knockdown activated cGAS/STING signalling pathway in NSCLC.

### 3.7 SOX2 inhibited cGAS/STING signalling via regulating cGAS transcription

**FIGURE 5:**
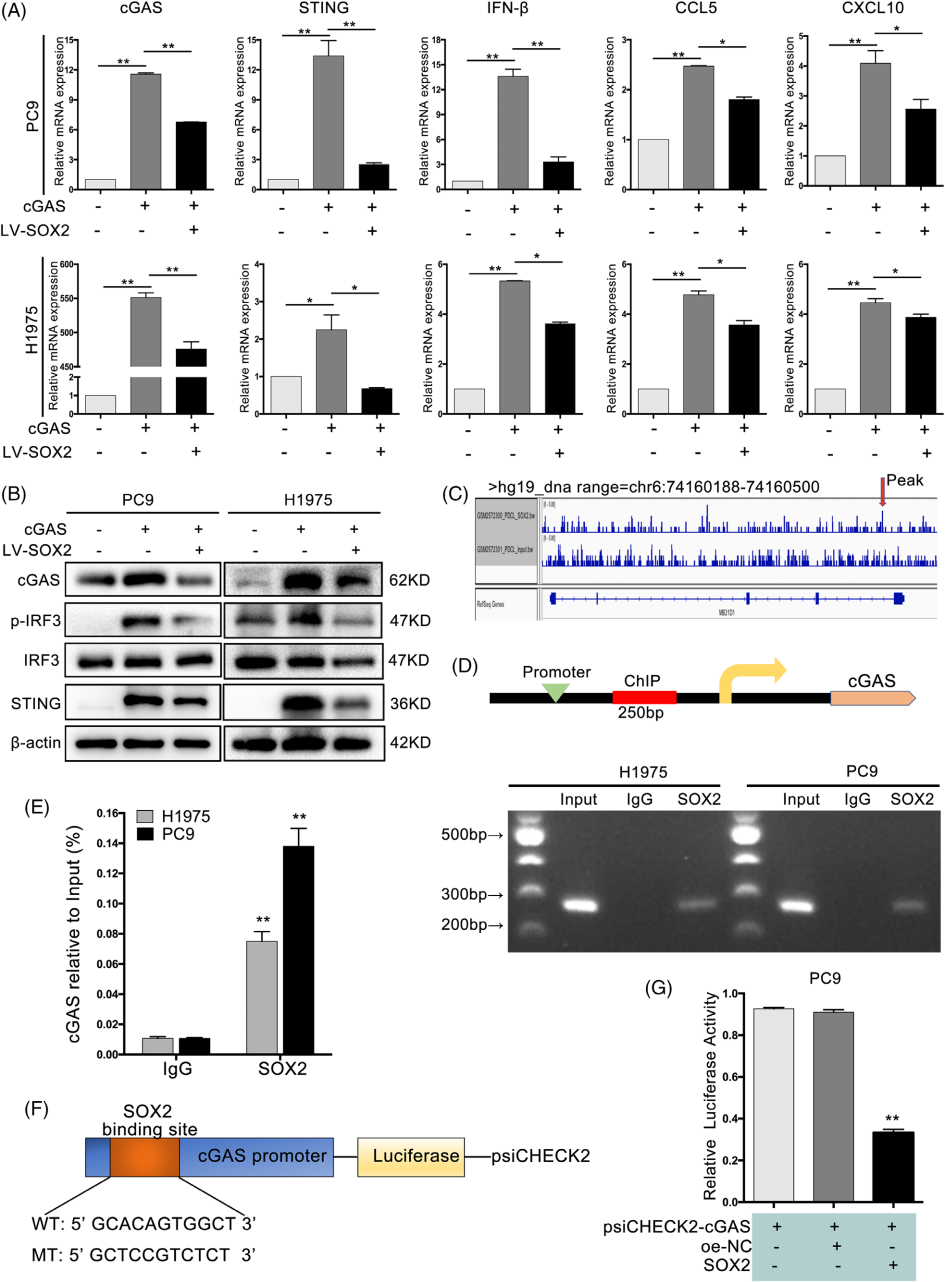
Sex‐determining region Y‐related high‐mobility group box 2 (SOX2) inhibited cGAS/STING signalling by direct trans‐repression of cGAS and STING expression. (A) The mRNA levels of cGAS, STING, IFNβ, CCL5 and CXCL10 in non‐small cell lung cancer (NSCLC) cells with cGAS and SOX2 overexpression. (B) Representative immunoblotting of cGAS, STING, p‐IRF3 and IRF3 in NSCLC cells with cGAS and SOX2 overexpression. (C) One putative SOX2 binding site is predicted using the integrative genomic viewer in cGAS promoter, as pointed by red arrow. (D) Chromatin immunoprecipitation (ChIP) assay shows the enrichment of SOX2 on the predicted promoter of cGAS and demonstrates the direct binding of SOX2 to the cGAS promoter in H1975 and PC9 cells. (E) Real‐time polymerase chain reaction (RT‐PCR) of the ChIP products confirmed the direct binding capacity of SOX2 to the cGAS promoter in H1975 and PC9 cells. (F) Schematic diagram of the luciferase reporter constructs incorporating wild‐type cGAS promoter and mutant cGAS promoter in which the presumed SOX2 binding site was deleted. (G) Luciferase reporter assay was performed to detect the luciferase activity after co‐transfected with psiCHEK2‐cGAS (WT or MT) and SOX2 overexpression. *p* = 3; *, *p* < .05; **, *p* < .01

As an important transcription factor, SOX2 can recognise and bind to the promoter of numerous target genes through its special TAD domain in various physiological processes.^39^ To better understand how SOX2 regulated cGAS/STING signalling, we investigated whether SOX2 directly controlled the expression of cGAS. We first co‐transfected cGAS‐expressing and SOX2‐expressing plasmids in PC9 and H1975 cells. The mRNA levels of cGAS were notably inhibited in the presence of ectopic SOX2 expression. SOX2 potently suppressed STING, IFN‐β, CCL5 and CXCL10 expression in NSCLC cells, while cGAS could partially rescue them (Figure 5A). Moreover, SOX2 inhibited STING expression and IRF3 phosphorylation in NSCLC cells, while cGAS substantially reversed these impacts (Figure 5B). Besides, we uncovered that the cGAS promoter harboured one potential SOX2 binding site (chr6:74160188‐74160500) through the predictor tool Integrative Genomics Viewer (Figure 5C). The ChIP assay demonstrated the evident enrichment of SOX2 in the predicted region of the cGAS promoter (Figure 5D). This was further verified by ChIP‐qPCR assays in PC9 and H1975 cells (Figure 5E). Furthermore, the promoter region of cGAS that contained SOX2 motif was cloned into a luciferase reporter plasmid as psiCHECK2‐cGAS, and the binding sequence of SOX2 motif was mutated from GACAGTGGCT to GCTCCGTCTCT in the psiCHECK2‐SOX2‐mutant plasmid (Figure 5F). Co‐expression of SOX2 resulted in significant repression of the cGAS promoter (Figure 5G). These results suggested that PCAT1 inhibited cGAS/STING pathway via upregulating SOX2 to directly repress cGAS transcription.

### 3.8 PCAT1 promoted tumour progression and restricted cGAS/STING signalling via modulating SOX2 in NSCLC

**FIGURE 6:**
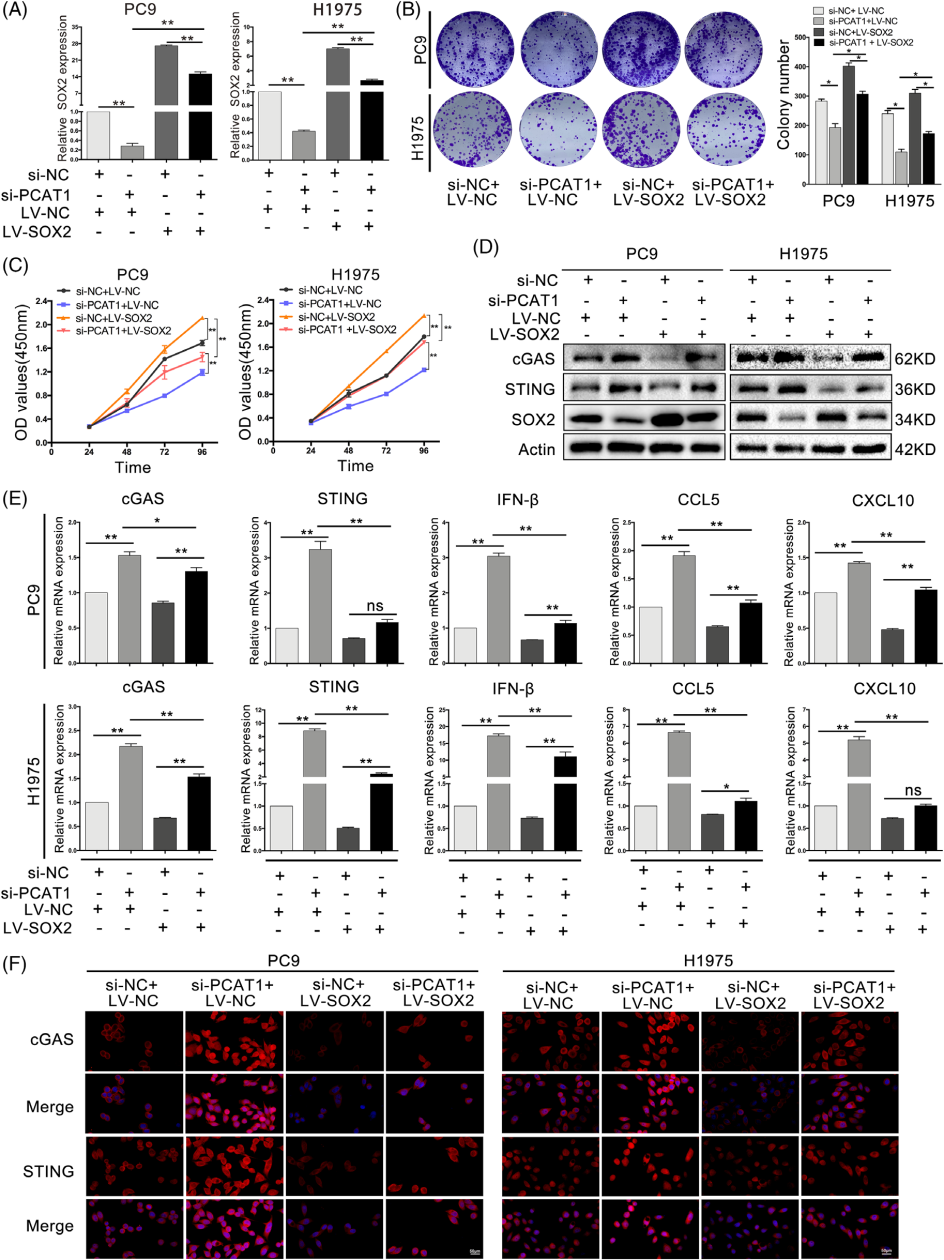
Prostate cancer‐associated ncRNA transcripts 1 (PCAT1) promoted non‐small cell lung cancer (NSCLC) cell growth and restricted cGAS/STING signalling via modulating sex‐determining region Y‐related high‐mobility group box 2 (SOX2). (A) The mRNA levels of SOX2 in PC9 and H1975 cells with si‐PCAT1 and SOX2 plasmid treatments. (B) Colony formation of PC9 and H1975 cells with si‐PCAT1 and SOX2 plasmid treatments. (C) Cell counting kit‐8 (CCK8) assays of NSCLC cells with si‐PCAT1 and SOX2 plasmid treatments. (D) Representative immunoblotting of SOX2, cGAD and STING in NSCLC cells with si‐PCAT1 and SOX2 plasmid treatment. (E) The mRNA levels of cGAS, STING, IFNβ, CCL5 and CXCL10 in PC9 and H1975 cells with si‐PCAT1 and SOX2 plasmid treatments. (F) Representative immunofluorescence of cGAS and STING protein in PC9 and H1975 cells with si‐PCAT1 and SOX2 plasmid treatments. *n* = 3; **, *p* < .01; ***, *p* < .001

To verify whether PCAT1 restricted cGAS/STING signalling through regulation of SOX2 activity, we stably co‐transfected PC9 and H1975 cells with si‐PCAT1 and SOX2‐expressing lentiviruses. Exogenous SOX2 expression partially rescued si‐PCAT1‐suppressed SOX2 expression (Figure 6A). Furthermore, SOX2 partly reversed the restrictive effects of si‐PCAT1 on NSCLC cell colony formation and proliferation (Figure 6B,C). Moreover, SOX2‐expressing lentiviruses partially significantly reversed cGAS and STING expression in PCAT1‐deficient NSCLC cells (Figure 6D), and SOX2 reversed the mRNA levels of cGAS/STING pathway components inhibited by PCAT1 silencing (Figure 6E). Immunofluorescence suggested the similar rescue effects of SOX2 in PCAT1‐deficient NSCLC cells (Figure 6F). These results indicated that SOX2 mediated the impacts of PCAT1 on cell behaviours in NSCLC cells.

### 3.9 Inhibition of PCAT1/SOX2 enhanced IR‐induced cGAS/STING activation and immunomodulatory potential

**FIGURE 7:**
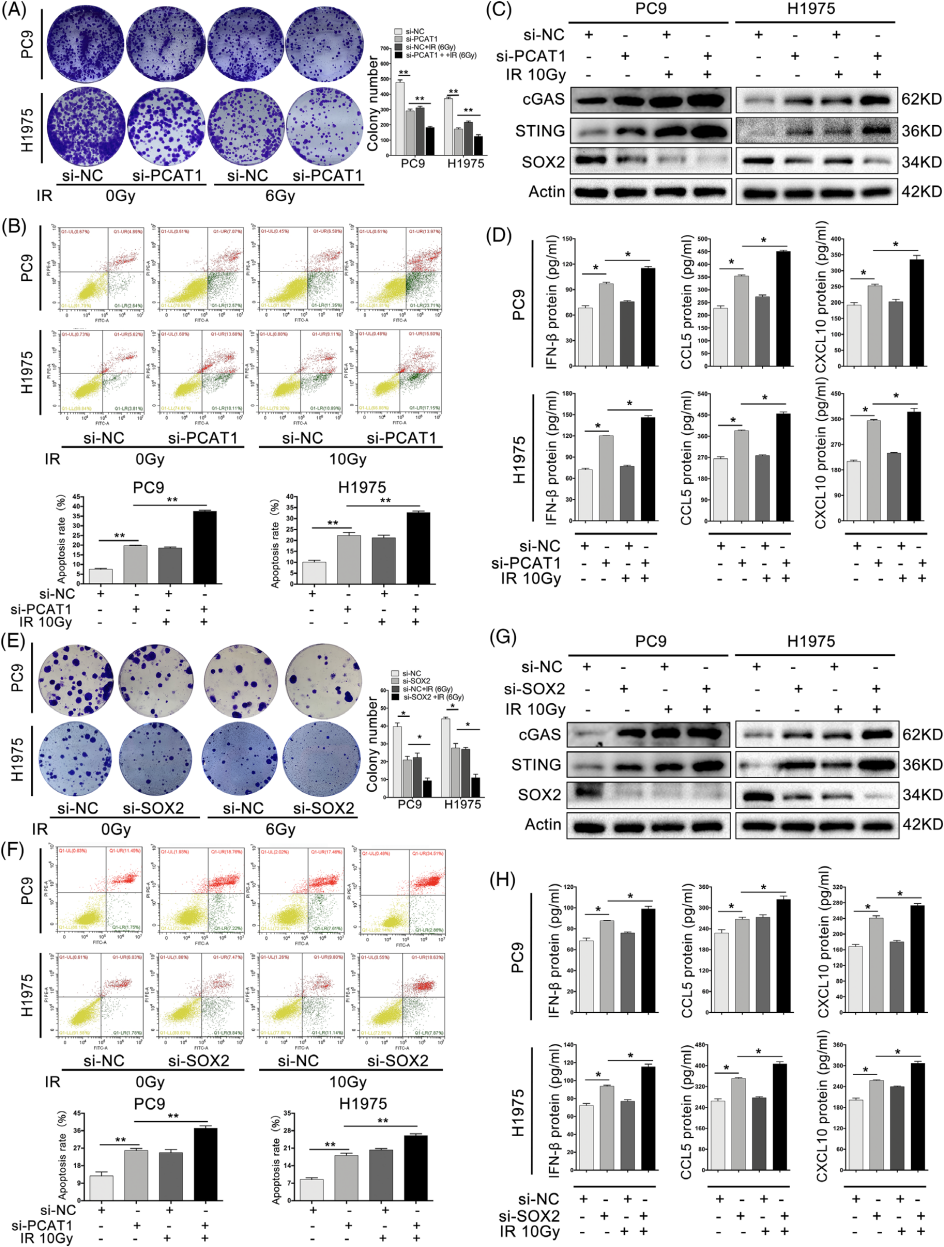
Inhibition of prostate cancer‐associated ncRNA transcripts 1/sex‐determining region Y‐related high‐mobility group box 2 (PCAT1/SOX2) enhanced ionising radiation (IR)‐induced cGAS/STING activation and anti‐tumour immune responses. (A) The synergistic effects of si‐PCAT1 and IR (6 Gy) on PC9 and H1975 cell colony formation. (B) The synergistic effects of si‐PCAT1 and IR (10 Gy) on non‐small cell lung cancer (NSCLC) cell apoptosis. (C) Representative immunoblotting of SOX2, cGAS and STING proteins in PC9 and H1975 cells with si‐PCAT1 and IR (10 Gy). (D) The synergistic effects of si‐PCAT1 and IR (10 Gy) on the secretion of IFNβ, CCL5 and CXCL10 in PC9 and H1975 cells. (E) The synergistic effects of si‐SOX2 and IR (6 Gy) on PC9 and H1975 cell colony formation. (F) The synergistic effects of si‐SOX2 and IR (10 Gy) on NSCLC cell apoptosis. (G) Representative immunoblotting of SOX2, cGAS and STING proteins in PC9 and H1975 cells with si‐SOX2 and IR (10 Gy). (H) The synergistic effects of si‐PCAT1 and IR (10 Gy) on the secretion of IFNβ, CCL5 and CXCL10 in PC9 and H1975 cells. *n* = 3; *, *p *< .05; **, *p *< .01

It has been now well recognised that IR not only induced DNA damage and cell death in tumour cells, but also shapes innate immune responses in an IFN‐I‐dependent manner that facilitates adaptive immune responses.^40^ IR was reported to induce type I IFN,^41^, ^42^ however, the regulatory effects of PCAT1 and SOX2 in this process were still be to investigated. We examined whether PCAT1/SOX2 contributed to cGAS/STING activation following IR. First, it was attested that PCAT1 and SOX2 knockdown could enhance radiosensitivity of NSCLC cells in vitro. PCAT1 silencing and IR (6 Gy) inhibited NSCLC cell colony formation in a collaborative manner (Figure 7A). IR at 6 Gy was used for colony formation assay, because 10 Gy was too strong for both PC9 and H1975 cells at low density for this experiment (Figure S12). NSCLC cell apoptosis was further enhanced in the si‐PCAT1 plus 10 Gy IR group (Figure 7B). The expression of cGAS, STING, IFN‐β, CCL5 and CXCL10 was raised by 10 Gy IR (Figure S13A). Moreover, IR induced cGAS/STING signalling pathway in a time‐dependent manner, and we chose 48 h after radiotherapy as the sampling time for subsequent experiments (Figure S13B). Immunoblotting and enzyme‐linked immunosorbent assay (ELISA) proved that PCAT1 depletion and IR (10 Gy) further elevated the cGAS and STING protein levels and induced IFN‐β, CCL5, CXCL10 in NSCLC cells (Figure 7C,D). PCAT1 activity was associated with IR responses through modified GSEA (Figure S13C), and PCAT1 expression was gradually decreased in a dose‐dependent manner (Figure S13D). GSEA also associated SOX2 expression with DNA repair (Figure S13E). The mRNA and protein levels of SOX2 were gradually decreased in a dose‐dependent manner (Figure S13F,G). SOX2 knockdown and IR had cooperating effects on colony formation and apoptosis (Figure 7E,F). Similar results were obtained with downregulation of SOX2 in collaboration with IR for cGAS/STING pathway proteins in PC9 and H1975 cells (Figure 7G,H). These results revealed that downregulation of PCAT1/SOX2 and radiation synergistically amplified the immunomodulatory potential via activating the cGAS/STING signalling pathway.

### 3.10 Knockdown of PCAT1 inhibited NSCLC growth and increased radiosensitivity in vivo

**FIGURE 8:**
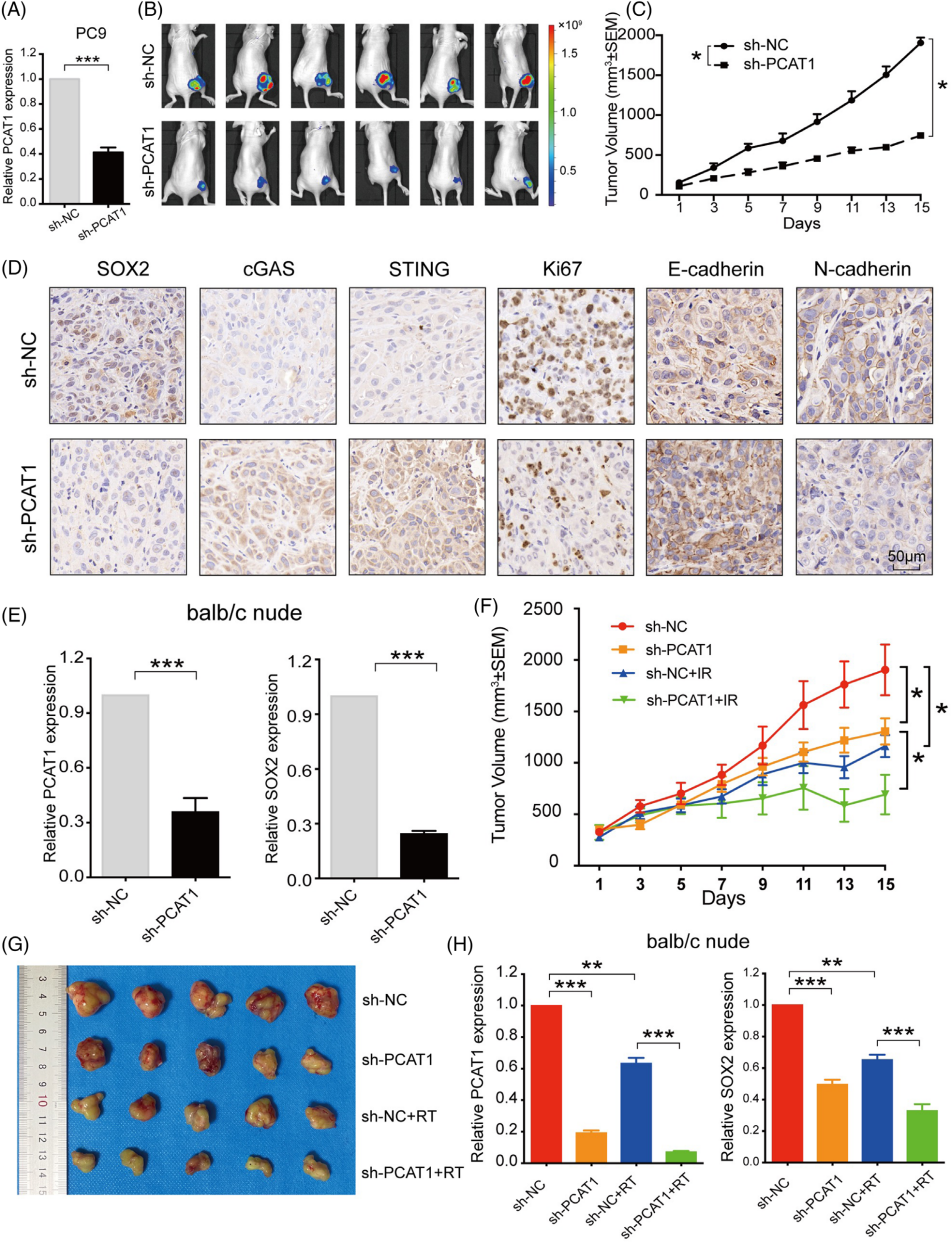
Prostate cancer‐associated ncRNA transcripts 1 (PCAT1) knockdown inhibited non‐small cell lung cancer (NSCLC) growth and increased radiosensitivity in vivo. (A) The efficiency of sh‐PCAT1 was evaluated in PC9 cells. (B) Nude mice were injected with sh‐NC and sh‐PCAT1 lentivirus‐transinfected PC9 cells. The representative luciferase signals in the sh‐PCAT1 group were remarkably lower than those in the sh‐NC group. (C) The tumour volumes were measured every other day and depicted in the line chart. (D) Representative IHC of SOX2, cGAS, STING, Ki67, E‐cadherin and N‐cadherin in tumour tissues. (E) sh‐PCAT1 significantly downregulated PCAT1 and SOX2 expression in tumour tissues. (F) sh‐PCAT1 had synergic effects on tumour growth in vivo with ionising radiation (IR) (8 Gy * 3). (G) Representative photos of tumour xenografts 14 days after the initiation of IR. (H) Ionising radiation (IR) downregulated PCAT1 and SOX2 expression in the tumour tissues, and sh‐PCAT1 further decreased the mRNA levels reduced by IR. *n* ≥ 6; *, *p *< .05; ***, *p *< .001

To explore the potential of targeting PCAT1 in NSCLC, we established NSCLC tumour xenografts with PCAT1 stably depleted cells. The efficiency of PCAT1 depletion was validated by qRT‐PCR (Figure 8A). PCAT1 depletion led to significant retard in the growth of the NSCLC tumours (Figure 8B,C). In addition, Ki67, SOX2 and N‐cadherin were significantly downregulated in the PCAT1‐depleted tumours, while cGAS, STING and E‐cadherin were upregulated (Figure 8D). Moreover, we checked the PCAT1 and SOX2 expression in the tumour tissues, and the quantitative real‐time polymerase chain reaction (qRT‐PCR) results suggested that PCAT1 depletion downregulated SOX2 in vivo as well (Figure 8E). To investigate the effects of PCAT1 on radiosensitivity, mice injected with PCAT1‐deficient PC9 cells were subjected to IR at 8 Gy for three times. Interestingly, PCATa downregulation had a significantly synergic effect with IR on tumour growth (Figure 8F,G). Further experiments indicated that PCAT1 depletion also had a cooperative effect with IT on SOX2 expression. These results suggested that PCAT1 knockdown suppressed NSCLC cell growth and promoted radiosensitivity in vivo.

### 3.11 SOX2 silencing activated T cell–related immune responses and the inactivation of cGAS/STING could in part rescue via combination cGAS inhibitor RU.521 in vivo

To ulteriorly explore the effects of SOX2 silencing on immune cell infiltration in vivo, the C57BL/6 mice were inoculated with SOX2‐deficient LLC cells. The downexpression of SOX2 was validated by qRT‐PCR (Figure S14A). In this C57BL/6 xenograft model, SOX2 absence also notably repressed tumour growth (Figure S14B). The SOX2 silencing group's tumour size was lesser than that in the LV‐sh‐NC group (Figure S14C). Meanwhile cGAS inhibitor partly impaired the induction of cGAS/STING signalling induced by SOX2 downregulation. Cytotoxic T cells were analysed in tumour tissues by means of flow cytometry. SOX2 silencing augmented CD8+ T cells in tumours, and CD8+ T cells declined in part after the addition of cGAS inhibitor RU.521 (Figure S14D,E). Next, IHC was used to examine the expression of SOX2, cGAS, STING, ki67, CD3 and CD8 in tumours. The consequences were accordant with flow cytometry analysis (Figure S14F). In summary, downregulation of SOX2‐activated T cell‐related immune responses, and the induction of cGAS/STING signalling induced by SOX2 downregulation was partially impaired when cGAS was depleted.

### 3.12 SOX2 overexpression dampened IR‐induced adaptive immune responses in vivo

**FIGURE 9:**
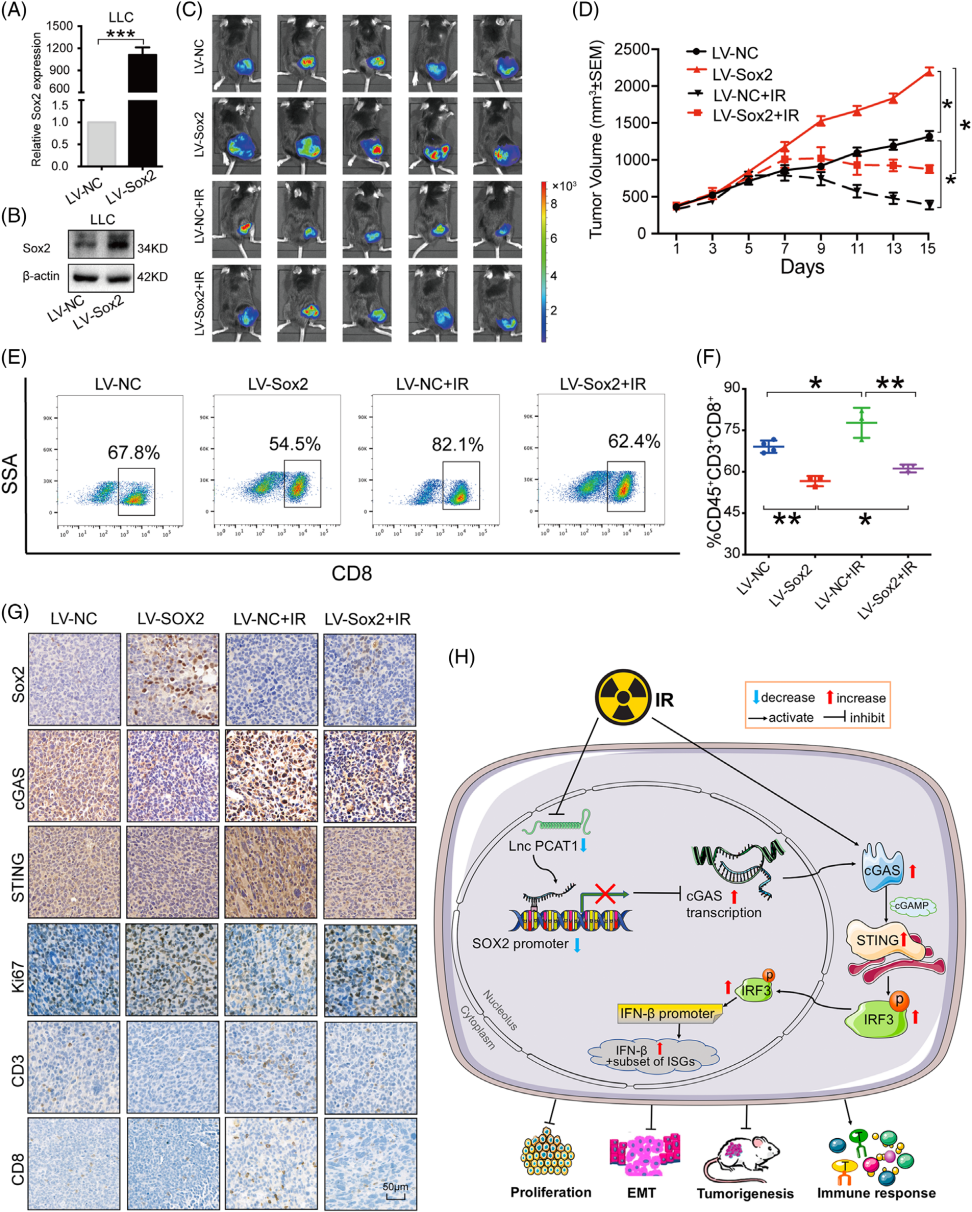
Sex‐determining region Y‐related high‐mobility group box 2 (SOX2) overexpression diminished radioimmune responses induced by ionising radiation (IR). (A) The efficiency of LV‐SOX2 was evaluated in Lewis lung carcinoma (LLC) cells. (B) LV‐SOX2 increased the protein levels of SOX2 in LLC cells. (C) C57B/L mice were injected with LV‐NC and LV‐SOX2 lentivirus‐transinfected LLC cells, and subjected to bioluminescent imaging and representative intravascular ultrasound spectrum imaging for tumour‐bearing mice. (D) The tumour volumes were measured every other day and depicted in the line chart. (E) Representative flow cytometry of CD8+ T cells in the tumour tissues. (F) Quantification of CD8+ T cells in the tumor microenvironment (TME) from mice with LV‐SOX2 and IR treatments. (G) Representative immunohistochemistry (IHC) images of SOX2, cGAS, STING, Ki67, CD3 and CD8 in tumour tissues. Scale bar, 50 μm. (H) A schematic model of the mechanism underlying that PCAT1 activates SOX2 and suppresses radioimmune responses via regulating cGAS/STING signalling in NSCLC. *n* ≥ 6; *, *p* < .05; **, *p* < .01; ***, *p* < .001

To confirm the mediatory effects of SOX2 in radioimmune responses in vivo, we overexpressed SOX2 in LLC cells and injected them into immune‐competent C57B/L mice. The overexpression of SOX2 was validated by qRT‐PCR and immunoblotting (Figure 9A,B). SOX2 upregulation impaired the inhibitory effects of IR on tumour growth (Figure 9C,D). Flow cytometry revealed decreased CD8+ T cells in the TME (Figure 9E,F), suggesting diminished radioimmune responses. The results of IHC exhibited that the expression of CD3, CD8, cGAS and STING were reduced in SOX2‐expressing tumours (Figure 9G). Collectively, SOX2 upregulation dampened intrinsic host immune activation of cGAS /STING signalling, and inhibited IR‐induced anti‐tumour immune responses.

## 4 DISCUSSIONS

In this study, we related PCAT1/SOX2 axis to NSCLC radioimmune responses. First, we found that PCAT1 induced SOX2 expression via directly regulating SOX2 promoter region. Next, our findings demonstrated that PCAT1 activated SOX2 to regulate cytotoxic T‐cell infiltration in NSCLC via inhibiting cGAS/STING pathway, thereafter rendering an immunosuppressive TME. Our in‐depth studies discovered that downregulation of PCAT1 could be combined with radiotherapy to activate the immune responses and achieve preferably NSCLC suppression. Therefore, valuable therapeutic targets for cGAS/STING pathway activation represented a potential strategy with major implications for anti‐tumour and cancer immunotherapy.

Previous studies implicated that PCAT1 was an oncogenic gene and played vital roles in the regulation of cancer development. Prensner JR et al. showed that higher expression of PCAT1 promoted prostate cancer cell proliferation through c‐Myc.^43^ PCAT1 overexpression promoted gastric cancer cell proliferation and metastasis via regulating CDKN1A.^44^ Shang Z et al. found that PCAT1 activated AKT and NF‐κB via regulating the PHLPP/FKBP51/IKK complex in prostate cancer.^45^ Several studies in recent years have also found important roles of PCAT1 in lung cancer. It was demonstrated that PCAT1 promoted NSCLC progression via regulating miR‐149‐5p/LRIG2 axis.^21^ Recent studies presented that PCAT1 also interacted with DKC1 to regulate NSCLC cell proliferation and apoptosis through the VEGF/AKT/Bcl‐2/caspase9 pathway.^46^ The mechanisms of lncRNA function in various cellular contexts are very complicated, including post‐transcriptional regulation, organisation of protein complexes and cell‐cell signaling.^47^ LncRNAs regulate the functions of multiple cells, genes and pathways involved in innate and adaptive immunity.^48^, ^49^ The present study demonstrated the roles of PCAT1 in mediating cGAS/STING signalling pathway through positive regulation of SOX2, affecting radioimmunity in NSCLC. More importantly, in a cohort of 55 NSCLC patients, the PCAT1 expression was positively correlated with SOX2 in NSCLC tissues. Our discoveries provided original insight into exploring the regulatory mechanisms of PCAT1 in NSCLC. Our studies indicated that PCAT1 knockdown increased radiosensitivity in NSCLC cells, as similar impact on chemosensitivity previously reported.^50^ Its inhibitory effects on cGAS/STING signalling pathway might serve as effective biomarkers for cancer immunotherapy and represent potential therapeutic strategies for NSCLC patients.

LncRNAs modulate transcription via altering nuclear structures, histone modifications and signalling complexes.^51^ Our studies focussed on its regulation of SOX2 activity and the underlying mechanisms. Several transcription factors are associated with delicately regulated lncRNAs within their promoters, resulting in the modulation of their transcriptional activities.^52^, ^53^, ^54^ In particular, we discovered how PCAT1 regulated the transcriptional activity of SOX2. Mechanistically, PCAT1 binds to SOX2 promoter, leading to accelerated SOX2 transcription. SOX2 mRNA and protein levels were precisely regulated at the transcriptional, post‐transcriptional and post‐translational levels.^55^ Multiple lncRNAs were reported to regulate SOX2 expression at the post‐transcriptional level. SOX2OT was reported to upregulate SOX2 substantially and augment breast cancer cell growth,^56^ whereas knockdown of SOX2OT impeded SOX2 transcription in bladder cancer.^57^ Tcl1 upstream neuron‐associated lncRNA was reported to induce SOX2 via recruiting 3 RNA‐binding proteins (PTBP1, hnRNPK and NCL) to the SOX2 promoter.^58^ Our study confirmed that PCAT1 positively regulated SOX2 expression by direct regulation of SOX2 mRNA levels. Thus, the underlying mechanism how PCAT1 induced SOX2 remains to be determined.

SOX2 amplification or overexpression is positively associated with advanced tumorigenesis, drug resistance and poor prognosis. SOX2 regulates various biological processes, containing apoptosis, cell‐cycle and autophagy, via modulating multiple signalling pathways.^59^ Zhong F et al. discovered that transcriptional activation of PD‐L1 by SOX2 contributed to the proliferation and caused adaptive immune resistance in hepatocellular carcinoma cells.^60^ Recent studies uncovered that SOX2 triggered resistant to T cell‐mediated cytotoxicity and anti‐PD‐1 with PD‐L1 high expression in melanoma.^61^ Recently, a variety of studies have validated that cGAS‐STING pathway played critical roles in type I IFN signalling and anti‐cancer immunity. Activated cGAS‐STING pathway and type I IFN signal were essential for tumour‐specific T‐cell cross‐priming and the whole cancer‐immunity cycle.^62^ cGAS is a significant cytosolic DNA sensor that triggers STING‐dependent signal and induces immune responses via further producing cGAMP, which stimulated the STING‐TBK1‐IRF3 signalling pathway to activate transcription of downstream factors embodying IFNβ, CCL5 and CXCL10.^63^ SOX2 was reported to promote the degradation of STING protein in an autophagy‐dependent manner in cancer cells.^24^ To investigate whether SOX2 also downregulated STING and downstream genes in NSCLC by this manner, autophagy inhibitor bafilomycin A1 was used to treat SOX2‐overexpression NSCLC cells. Our results suggested that SOX also downregulated cGAS/STING and downstream genes in an autophagy‐dependent manner (Figure S15).

Several investigations found that radiotherapy was significant in the clinic for its ability to induce anti‐tumour T cells and boost responses to immune checkpoint inhibitors and other immunotherapies.^64^, ^65^ Previous studies demonstrated that the expression of cGAS and STING, as well as their function in the cancer cells, were essential for optimal anti‐tumour T cell induction by radiotherapy.^66^ We observed that PCAT1 and SOX2 were downregulated in NSCLC after radiation, and that inhibiting their expression in collaboration with IR further activated the cGAS/STING signalling pathway. Meanwhile, we further demonstrated that immunosuppressive functions of SOX2 on NSCLC and T‐cell infiltration were partially weakened when collaborated with IR in vivo. However, further verification for tumour immune effects of intervention PCAT1 combined with radiotherapy was objectively restricted in vivo.

There were several limitations in our studies. First, BEAS‐2B cells are used as the “normal” lung cells to compare with NSCLC cancer lines. However, these cells are immortalised and have MSC‐like features.^67^ We will use actually normal primary cells in our future researches. Moreover, PCAT1 was difficult to be used to diagnose or in therapy of NSCLC, owing to the astriction of lncRNA detection. The roles of targeted molecules and combined radiotherapy on immunity has received increasing attention, but its effects and underlying mechanism are currently unclear. The involvement of IR in the decreased expression of immunosuppressive molecules is still worth exploring in NSCLC, and the results would assist us to better recognise the roles of stronger immune activation when combined with radiotherapy. It will be interesting to see how to optimally combine radiation and depleting activity of specific immunosuppressive molecules to achieve the better therapeutic benefits.

## 5 CONCLUSIONS

In summary, we demonstrated that PCAT1/SOX2 axis was correlated with tumorigenesis in NSCLC and involved in immunosuppression via restraining cGAS/STING pathways. Inhibition of PCAT1/SOX2 enhanced IR‐induced cGAS/STING activation and anti‐tumour immune responses. These outcomes provided the novel mechanistic insights into the pathogenesis of NSCLC, and might imply PCAT1/SOX2 as prognostic factors and potential therapeutic targets. Our studies suggested the potential roles of PCAT1/SOX2 as predictors of tumour responses to the combination of radiation and immune checkpoint inhibitors (Figure 9H).

## CONFLICT OF INTEREST

The authors declare no conflict of interest.

## Supporting information

Supporting Information
