# Supplementary material for: LncRNA PCAT1 activates SOX2 and suppresses radioimmune responses via regulating cGAS/STING signalling in non‐small cell lung cancer
Source: Clin Transl Med. 2022 Apr 12;12(4):e792. doi: 10.1002/ctm2.792 (PMC9005924; doi:10.1002/ctm2.792)
Supplement: Supplementary file 1 — Supporting Information [file CTM2-12-e792-s001.pdf]

SUPPLEMENTARY METHODS

Cell proliferation assay

Cell proliferation was measured using a cell counting kit-8 (CCK8) kit (Dojindo, Japan). Cells were seeded in 96-well plates. The optical density values were measured at a wavelength of 450 nm every 24 h using a multimodal plate reader (PE, USA).

Colony formation assay

Cells were trypsinized and suspended into 6-well plates. After incubation at 37 °C for 12 h, the cells were irradiated at 6 Gy. After 14 days, the cells were washed with PBS and fixed with 4% paraformaldehyde for 15 min. The colonies were stained with crystal violet for 20 min. The colonies were counted, and the results were reported as the relative colony number.

Wound healing assay

Cells were seeded into 6-well plates. After confluent, the cells were scratched with a sterile 200-μl pipette tip and washed with PBS gently. The width of the scratch gap was monitored under an inverted microscope and photographed at 0 h and 48 h. In terms of the difference between the original width of the wound and the width after cell migration was quantified.

Modified Boyden chamber assays

For the migration assay, cells (6×10^4) were resuspended in 200 μl serum-free medium and seeded in the upper chamber of 24-well transwell units (Corning, USA). For the invasion assay, cells were concentrated to 7×10^4 cells in 300 ul and seeded in the upper chamber coated with Matrigel. The RPMI 1640/DMEM medium containing 10% FBS were used as a chemo-attractant in the bottom chamber. After incubation for 24 h, cells at the other side of the membrane were fixed with 4% paraformaldehyde and stained with 0.1% crystal violet. Images of the stained cells were photographed.

ELISA

For the measurement of IFN-β, CCL5, CXCL10 production in PC9 and H1975 cells, supernatants were collected from the cell cultures in 6-well plates for 48 h. The levels of IFN-β, CCL5, CXCL10 were determined using a Bioswape ELISA kit according to the manufacturer's instructions.

Immunohistochemistry

Formalin-fixed paraffin-embedded tissue specimens were sectioned at 4 mm. Slides were deparaffinized by xylene and ethyl alcohol. The slides were then immersed into 100 μl of 3% hydrogen peroxide at room temperature for 10 min to block endogenous peroxidase activity. After washing with PBS for 3 times, the sections were incubated with 5% bovine serum albumin (Sigma-Aldrich, USA) for 30 min, followed by incubation with antibodies at 4 °C overnight. Finally, all sections were dehydrated in gradient concentrations of ethanol and xylene and were mounted with neutral gum.

Comet assay

Comet assays were performed using Comet Assay Kit (abcam, USA) following manufacturer's instructions. Pictures were photographed using a fluorescent microscope, and the comet tails were analyzed by Pect (CASP 1.2.3 beta 1). The percentage of comet tail DNA content (TDNA%) was quantified and graphed.

cGAS inhibitor treatment *in vivo*

The cGAS inhibitor RU.521 was purchased from MCE, which was dissolved in 10% DMSO and then was diluted with corn oil. The inhibitor was intraperitoneally injected into C57BL/6 mice with subcutaneous injection of stably SOX2-deficient LLC cells (1×10^6 cells in 100 ul PBS per mouse), at a dose of 10 mg/kg daily starting from Day 1 to the day tumors reached approximately 100-200 mm^3.

SUPPLEMENTARY TABLES

Table S1. Sequences of siRNAs.

Gene	Target sequence
si-PCAT1 #1 sense	5′- CAAAGGAUAUAAGAUGCAUTT-3′
si-PCAT1 #1 antisense	5′- AUGCAUCUUAUAUCCUUUGTT-3′
si-PCAT1 #2 sense	5′- CUGACGUCUUGCCAACUAATT-3′
si-PCAT1 #2 antisense	5′- UUAGUUGGCAAGACGUCAGTT-3′
si-PCAT1 #3 sense	5′- GAUGACGCAAAGGAACCUATT-3′
si-PCAT1 #3 antisense	5′- UAGGUUCCUUUGCGUCAUCTT-3′
si-SOX2 #1 sense	5′- CUCAUGAAGAAGGAUAAGUTT-3′
si-SOX2 #1 antisense	5′- ACUUAUCCUUCUUCAUGAGCG-3′
si-SOX2 #2 sense	5′- CCAUGGGUUCGGUGGUCAATT-3′
si-SOX2 #2 antisense	5′- UUGACCACCGAACCCAUGGTT-3′
si-cGAS #1 sense	5′- GGCUAUCCUUCUCUCACAUTT-3′
si-cGAS #1 antisense	5′- AUGUGAGAGAAGGAUAGCCTT-3′
si-cGAS #2 sense	5′- GCCUUCUUUCACGUAUGUATT-3′
si-cGAS #2 antisense	5′- UACAUACGUGAAAGAAGGCTT-3′

Table S2. Primer sequences used for amplification.

Gene	Sequences
PCAT1 F	5'-TGAGAAGAGAAATCTATTGGAACC -3′
PCAT1 R	5'-GGTTTGTCTCCGCTGCTTTA -3′
SOX2 F	5′-GCCGAGTGGAAACTTTTGTCG-3′
SOX2 R	5′-GGCAGCGTGTACTTATCCTTCT-3′
β-actin F	5′-GACCACCTTCAACTCCATCAT-3′
β-actin R	5′-CCTGCTTGCTAATCCACATCT-3′
cGAS F	5′-GCCCTGCTGTAACACTTCTTAT-3′
cGAS R	5′-GGATAGCCGCCATGTTTCTT-3′
STING F	5′-GCTGCTGTCCATCTATTTCTACT-3′
STING R	5′-GCCGCAGATATCCGATGTAATA-3′
IFN-β F	5′-TTGTTGAGAACCTCCTGGC-3′
IFN-β R	5′-TGACTATGGTCCAGGCACAG-3′
CCL5 F	5′-CGCTGTCATCCTCATTGCTA-3′
CCL5 R	5′-CCAGACTTGCTGTCCCTCTC-3′
CXCL10 F	5'- CTGTACGCTGTACCTGCATCA-3′
CXCL10 R	5′-TTCTTGATGGCCTTCGATTC-3′
Murine Sox2 F	5′-ACAGCATGTCCTACTCGCAG-3′
Murine Sox2 F	5′-ATGCTGATCATGTCCCGGAG-3′

Table S3. Antibodies used in this research.

Antibody	Company	Catalog number	Dilution
E-Cadherin	Proteintech	20874-1-AP	1:1000
N-Cadherin	Proteintech	22018-1-AP	1:1000
Vimentin	Proteintech	10366-1-AP	1:1000
SOX2	Cell Signaling Technology	2748	1:1000
SOX2	GeneTex	GTX101507	1:7000
cGAS	Cell Signaling Technology	4691	1:1000
cGAS	Proteintech	26416-1-AP	1:200
cGAS	ABclonal	A8335	1:100
p-IRF3	Cell Signaling Technology	37829	1:1000
IRF3	Proteintech	11312-1-AP	1:1000
STING	Proteintech	19851-1-AP	1:1000
γH2AX	ABclonal	AP0687	1:200
dsDNA	Abcam	ab27156	1:1000
Ki-67	Proteintech	27309-1-AP	1:200
GAPDH	Proteintech	10494-1-AP	1:5000
β-actin	Proteintech	60008-1-Ig	1:1000
Fluor®488 Donkey Anti-Rabbit IgG	Antgene	ANT024	1:100
Dylight 549 Goat Anti-Mouse IgG	Abbkin	A23310	1:100
HRP-conjugated Goat Anti-Rabbit IgG	Proteintech	SA00001-2	1:10000
HRP-conjugated Goat Anti-Mouse IgG	Proteintech	SA00001-1	1:10000

Table S4. Primer sequences used for CHIRP.

Gene	Sequences
PCAT1 F	5'-CCTCTAAGTGCCAGTGCAGG -3′
PCAT1 R	5'-CACCCTTTGACCCTTGGCAT -3′
SOX2 F	5′-GAGTTGGACAGGGAGATGGC-3′
SOX2 R	5′-CAACACTCTCTCACGCCCTT-3′
GAPDH F	5′-CAAATTCCATGGCACCGTCA-3′
GAPDH R	5′-GACTCCACGACGTACTCAGC-3′

Table S5. Differential expression of PCAT1 in NSCLC and adjacent tissues.

	n	PCAT1 expression		Chi-square Value	p value
		High	Low		
NSCLC	55	30	25	4.303	<0.001
Adjacent tissues	47	16	31		

Table S6. Correlation between PCAT1 expression and clinicopathological characteristics.

	Variables	PCAT1 expression		Total	χ^2	p value
		low	high			
Sex					2.518	0.113
	Female	11	19	30		
	male	14	10	24		
Age (year)					0.411	0.522
	<61	13	13	26		
	≥61	12	17	29		
Grade					0.011	0.916
	I/II	17	20	37		
	III	8	10	18		
					0.875	0.349
Tumor size	≤4cm	11	17	28		
	>4cm	14	13	27		
T stage					1.720	0.423
	T1	5	5	10		
	T2	9	16	25		
	T3/ T4	11	9	20		
N stage					1.304	0.254
	N0/Nx	12	19	31		
	N1/N2/ N3	13	11	24		
M stage					1.730	0.188
	M0	25	28	53		
	M1b	0	2	2		
TNM stage					0.000	1.000
	I/ II	15	18	33		
	III/IV	10	12	22		

Table S7. Differential expression of SOX2 in NSCLC and adjacent tissues.

	n	SOX2 expression		Chi-square Value	p value
		High	Low		
NSCLC	43	27	16	20.189	<0.001
Adjacent tissues	26	2	24		

Table S8. Correlation between SOX2 expression and clinicopathological characteristics.

	Variables	SOX2 expression		Total	χ^2	p value
		low	high			
Sex					0.127	0.721
	Female	12	12	24		
	male	10	8	18		
Age (year)					0.587	0.443
	<61	12	9	21		
	≥61	10	12	22		
Grade					0.803	0.370
	I/II	14	16	30		
	III	8	5	13		
Tumor size					1.896	0.169
	≤4cm	9	13	22		
	>4cm	13	8	21		
T stage					0.239	0.625
	T1/ T2a	12	13	25		
	T2b/ T3/ T4	10	8	18		
N stage					1.226	0.268
	N0/Nx	11	14	25		
	N1 /N2/ N3	11	7	18		
M stage					0.977	0.323
	M0	21	21	42		
	M1b	1	0	1		
TNM stage					2.794	0.095
	I/ II	7	12	19		
	III/IV	15	9	24		

SUPPLEMENTARY FIGURES

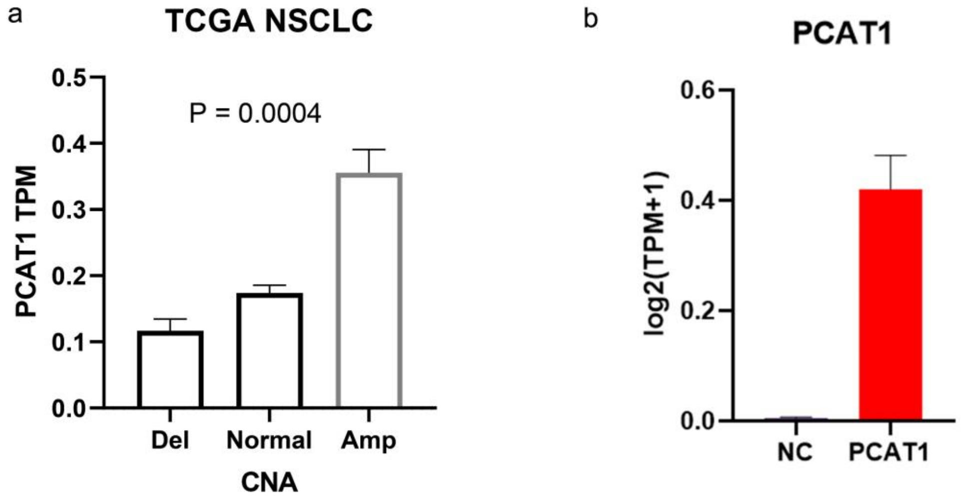

Figure S1. The relation of PCAT1 CNA with amplification and the efficiency of PCAT1 overexpression. (a) PCAT1 CNA was positively correlated with amplification in TCGA NSCLC. (b) Compared with negative control (NC), PCAT1-expressing plasmids significantly increased the expression levels of PCAT1 in PC9 cells for RNA-seq.

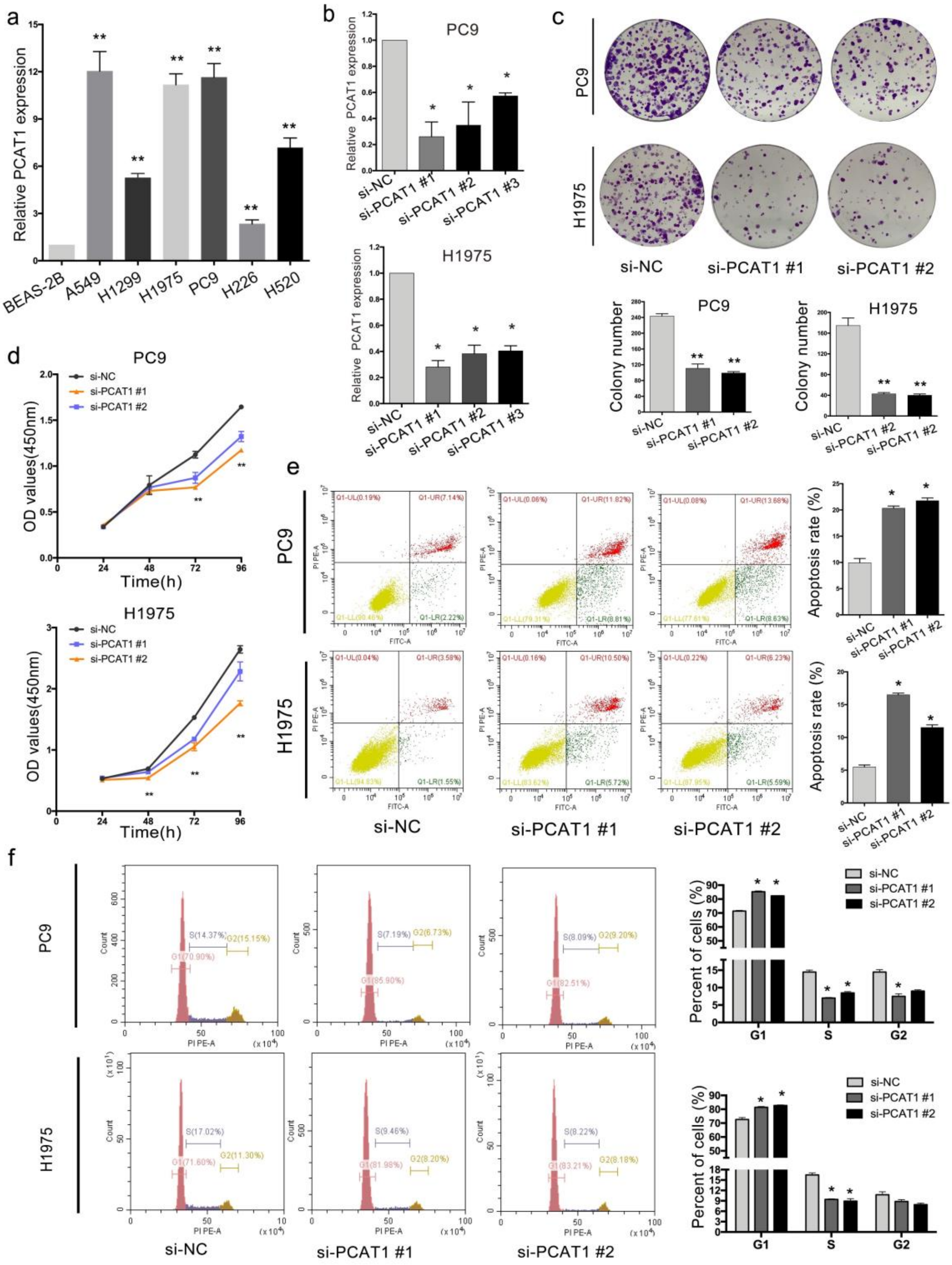

Figure S2. PCAT1 silencing inhibited NSCLC cell proliferation, increased apoptosis and induced S phase arrest. (a) qRT-PCR was performed to identify the expression levels of PCAT1 in 6 NSCLC cell lines and BEAS-2B cells. (b) The efficiency of si-PCAT1 was evaluated in NSCLC cells. (c) Colony forming assay indicated the decreased numbers of colonies after PCAT1 silencing in PC9 and H1975 cells. (d)

CCK8 assay showed that PCAT1 silencing significantly inhibited PC9 and H1975 cell proliferation. (e) NSCLC cell apoptosis was analyzed by flow cytometry 48 h after transfection. (f) NSCLC cell cycle was examined by flow cytometry 48 h after transfection. N = 3; *, P < 0.05; **, P < 0.01.

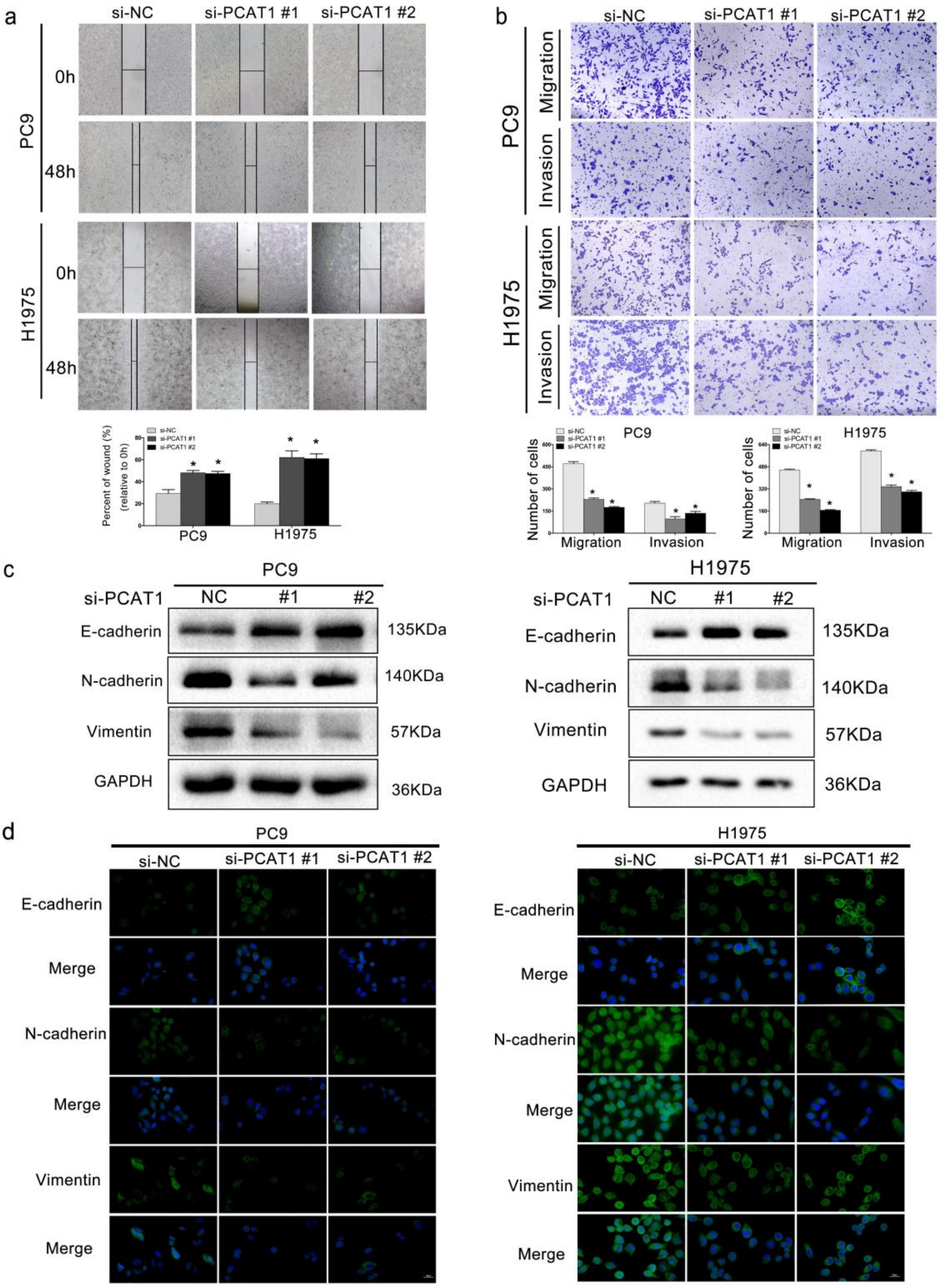

Figure S3. PCAT1 promoted NSCLC cell migration. (a) Wound healing showed that si-PCAT1 effectively decreased NSCLC cell motility. (b) PCAT1 downregulation inhibited NSCLC cell migration and invasion. (c) Representative immunoblotting of EMT markers in PC9 and H1975 cells after PCAT1 knockdown. (d) Representative

immunofluorescence of E-cadherin, N-cadherin and vimentin in PC9 and H1975 cells transfected with si-PCAT1. Scale bar, 200 μm. N = 3; *, P < 0.05.

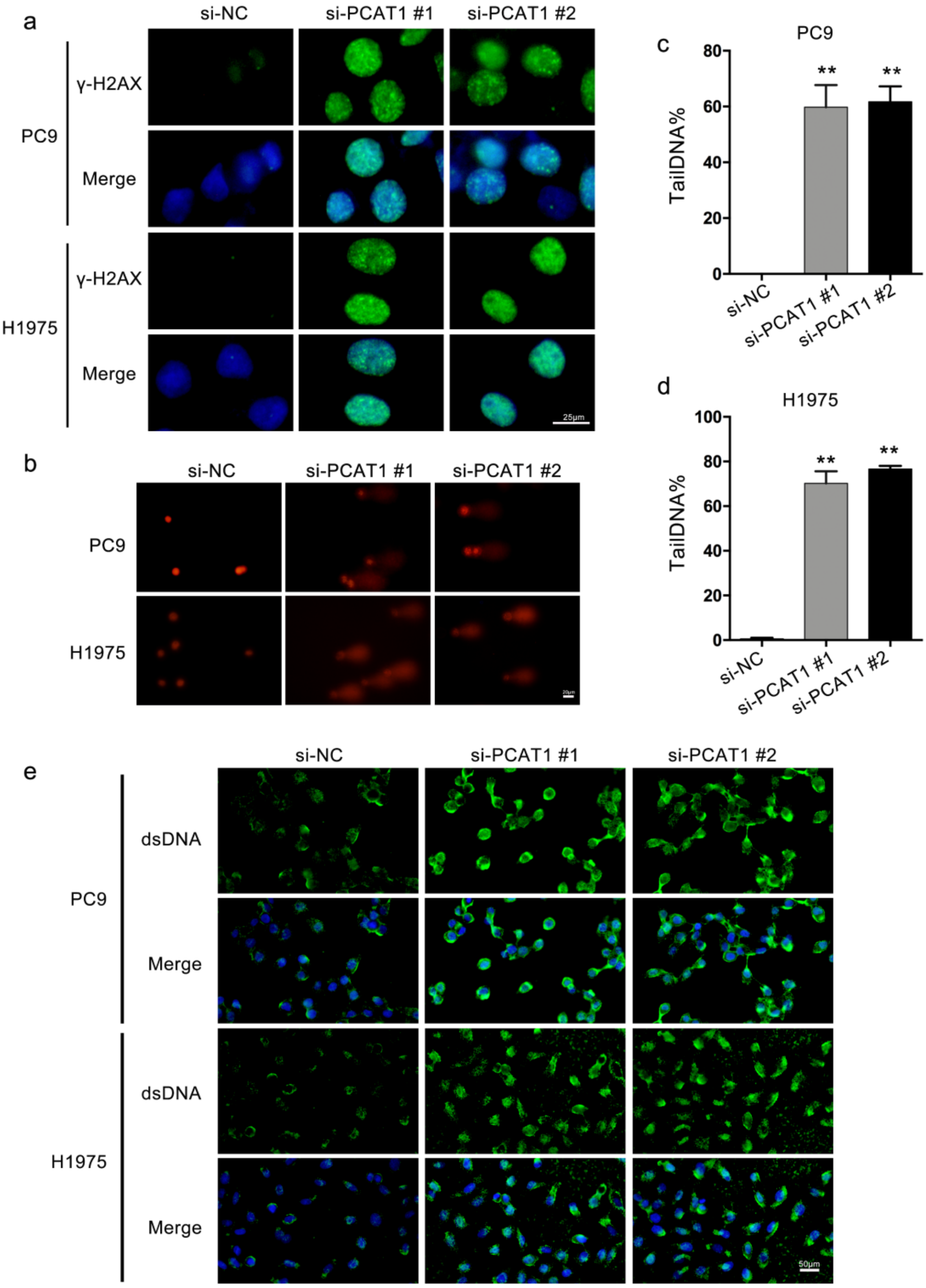

Figure S4. PCAT1 silencing induced DNA damage. (a) Representative immunofluorescence of γH2AX. Scale bar: 25 μm. (b) Representative Comet assay in

PCAT1-deficient PC9 and H1975 cells. Scale bar: 20 μm. (c, d) The percentage of tail DNA content was quantified. (e) Representative immunofluorescence of dsDNA in PCAT1-deficient PC9 and H1975 cells. Scale bar: 50 μm. N = 3; **, P < 0.01.

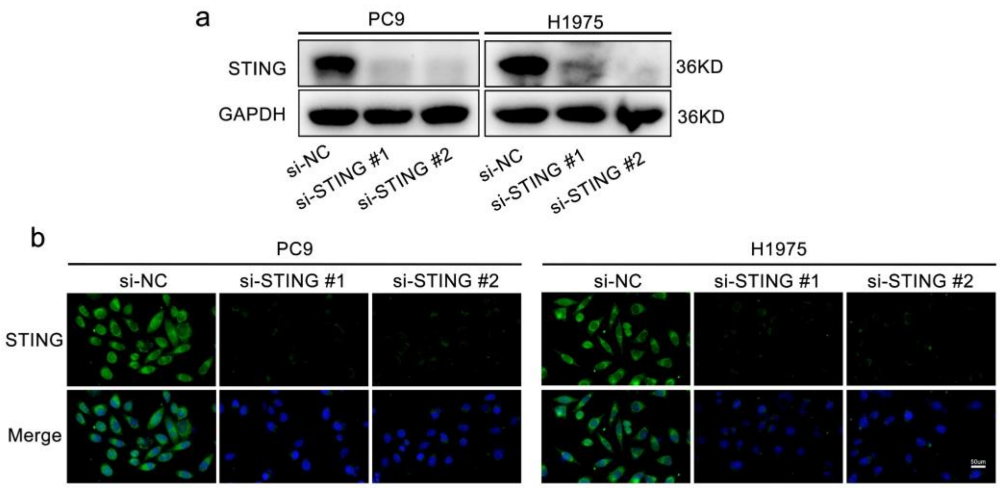

Figure S5. Immunofluorescence of STING in STING-deficient NSCLC cells were presented in to validate antibody staining. (a) Representative immunoblotting to validate the downregulation of PCAT1 by siRNAs. (b) Representative immunofluorescence of STING to validate the specific staining of antibodies.

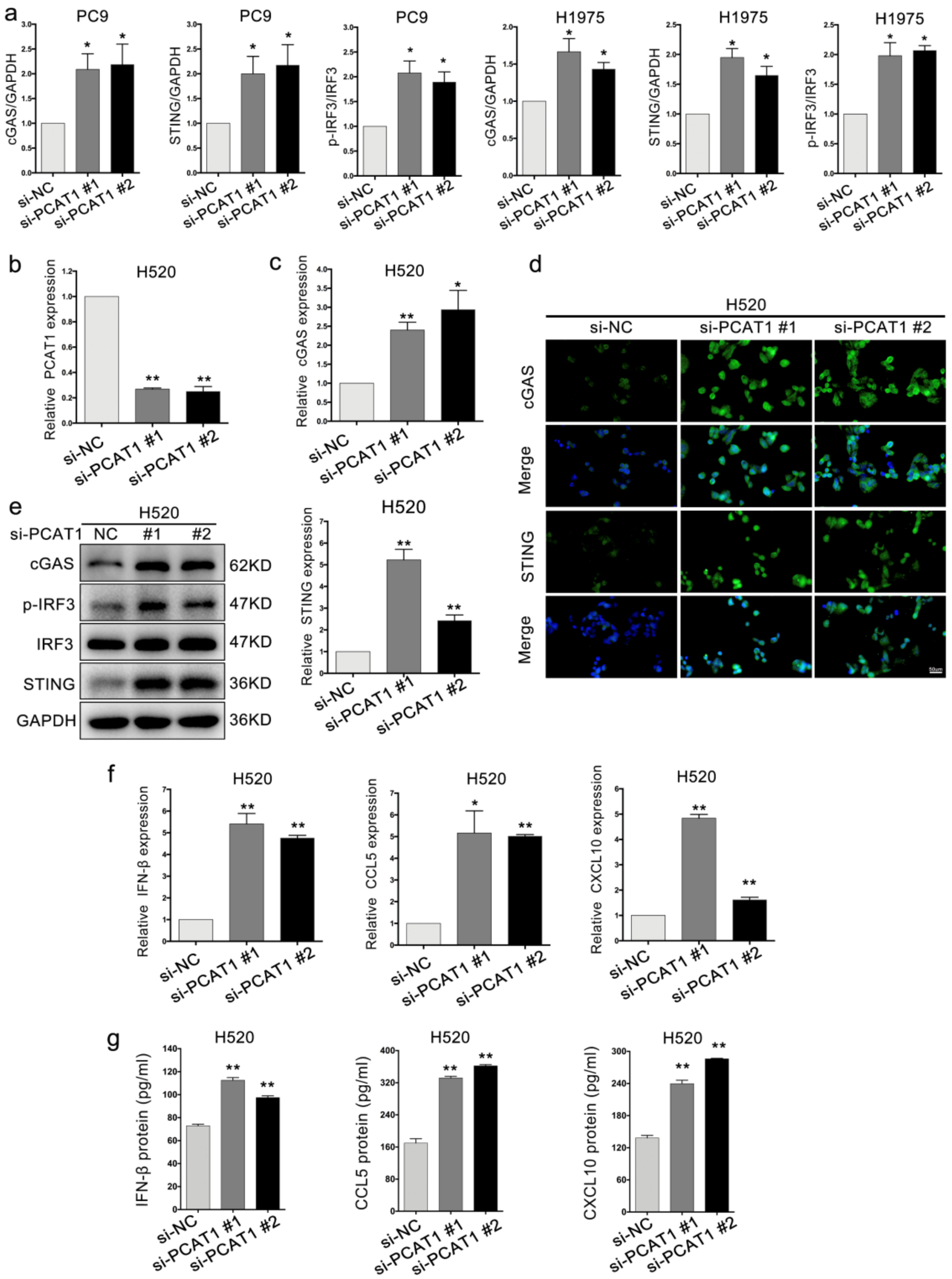

Figure S6. PCAT1 regulated cGAS/STING signaling pathway. (a) Quantification for immunoblotting of cGAS/STING pathway proteins in PCAT1-deficient PC9 and H1975 cells. (b) PCAT1 was downregulated by siRNAs in H520 cells. (c) PCAT1 knockdown increased sGAS mRNA levels in H520 cells. (d) Representative immunofluorescence of cGAS and STING in PCAT1-defecient H520 cells. (e)

PCAT1 depletion induced cGAS and STING protein levels, as well as IRF3 phosphorylation. (f) PCAT1 downregulation increased IFN-β, CCL5 and CXCL10 mRNA levels in H520 cells. (g) More IFN-β, CCL5 and CXCL10 were secreted by the PCAT1-deficient H520 cells. N = 3; *, $P < 0.05$; **, $P < 0.01$.

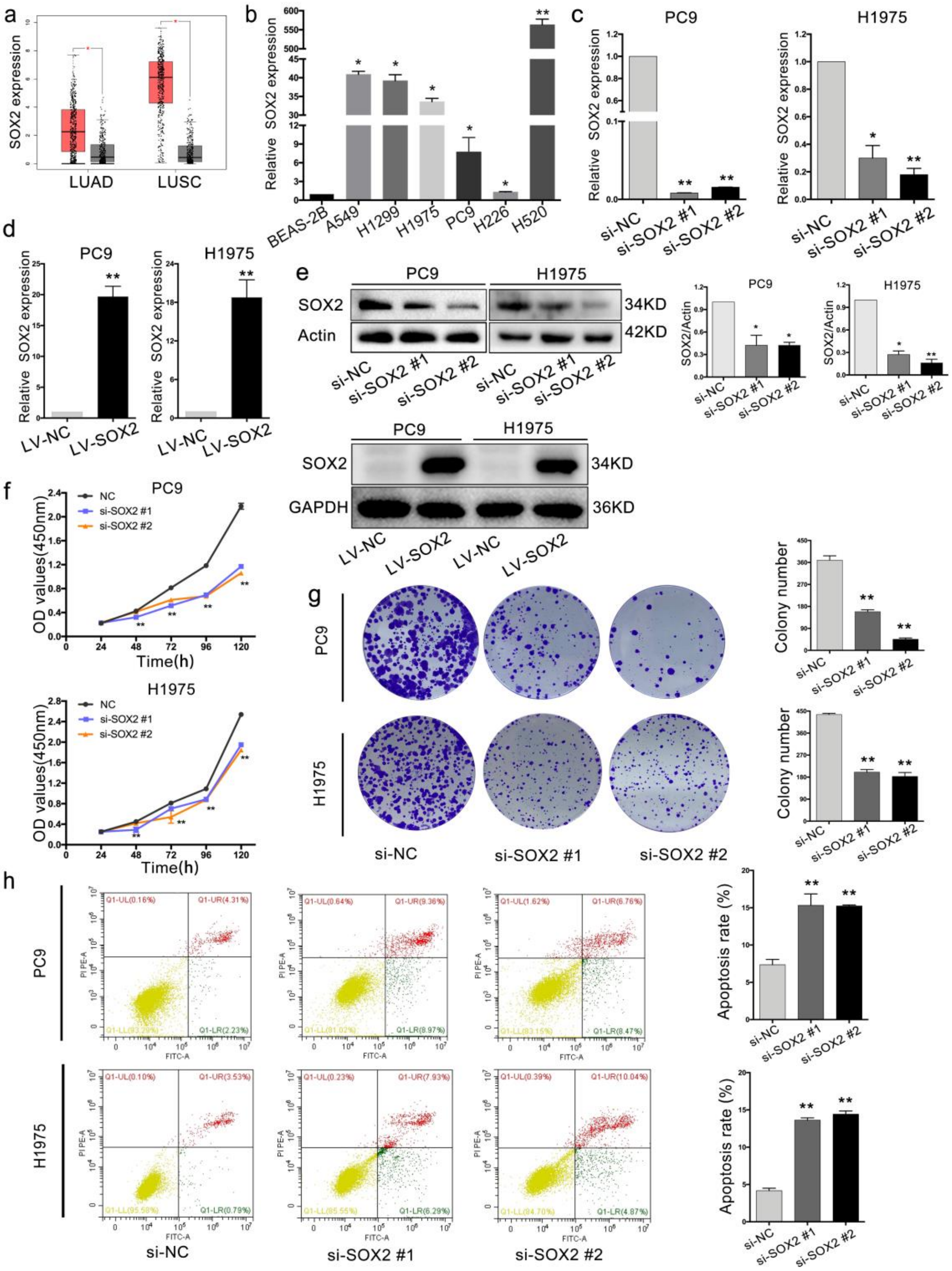

Figure S7. SOX2 knockdown inhibited NSCLC cell growth and induced apoptosis. (a) SOX2 was upregulated in both LUAD and LUSC samples. (b) The mRNA levels of SOX2 were higher in NSCLC cells. (c) SOX2 mRNAs were successfully downregulated by siRNAs in NSCLC cells. (d) SOX2 mRNAs were successfully upregulated by lentiviruses in NSCLC cells. (e) Representative immunoblotting of

SOX2 in NSCLC cells after si-SOX2 or LV-SOX2 treatments. (f) SOX2 knockdown suppressed NSCLC cell growth. (g) SOX2 knockdown inhibited NSCLC cell colony formation. (h) PC9 cells and H1975 cells were analyzed by flow cytometry for cell apoptosis 48 h after transfection. N = 3; *, $P < 0.05$; **, $P < 0.01$.

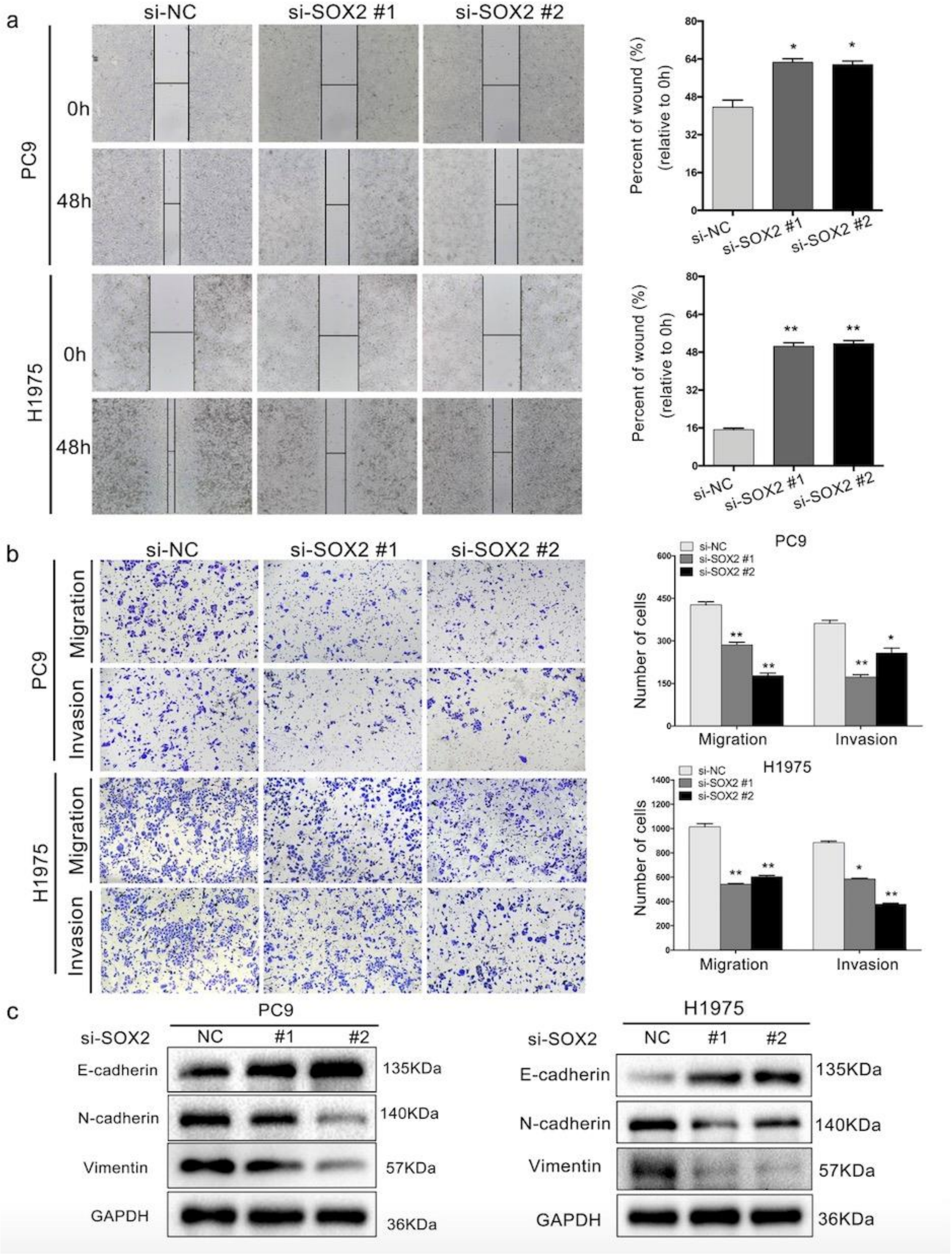

Figure S8. SOX2 knockdown inhibited NSCLC cell migration. (a) Wound healing assay showed that si-SOX2 significantly decreased NSCLC cell motility. (b) PCAT1 downregulation inhibited NSCLC cell migration and invasion. Scale bar, 200 μm. (c) Representative immunoblotting of E-cadherin, N-cadherin and vimentin in PC9 and H1975 cells after PCAT1 knockdown. N = 3; *, $P < 0.05$; **, $P < 0.01$.

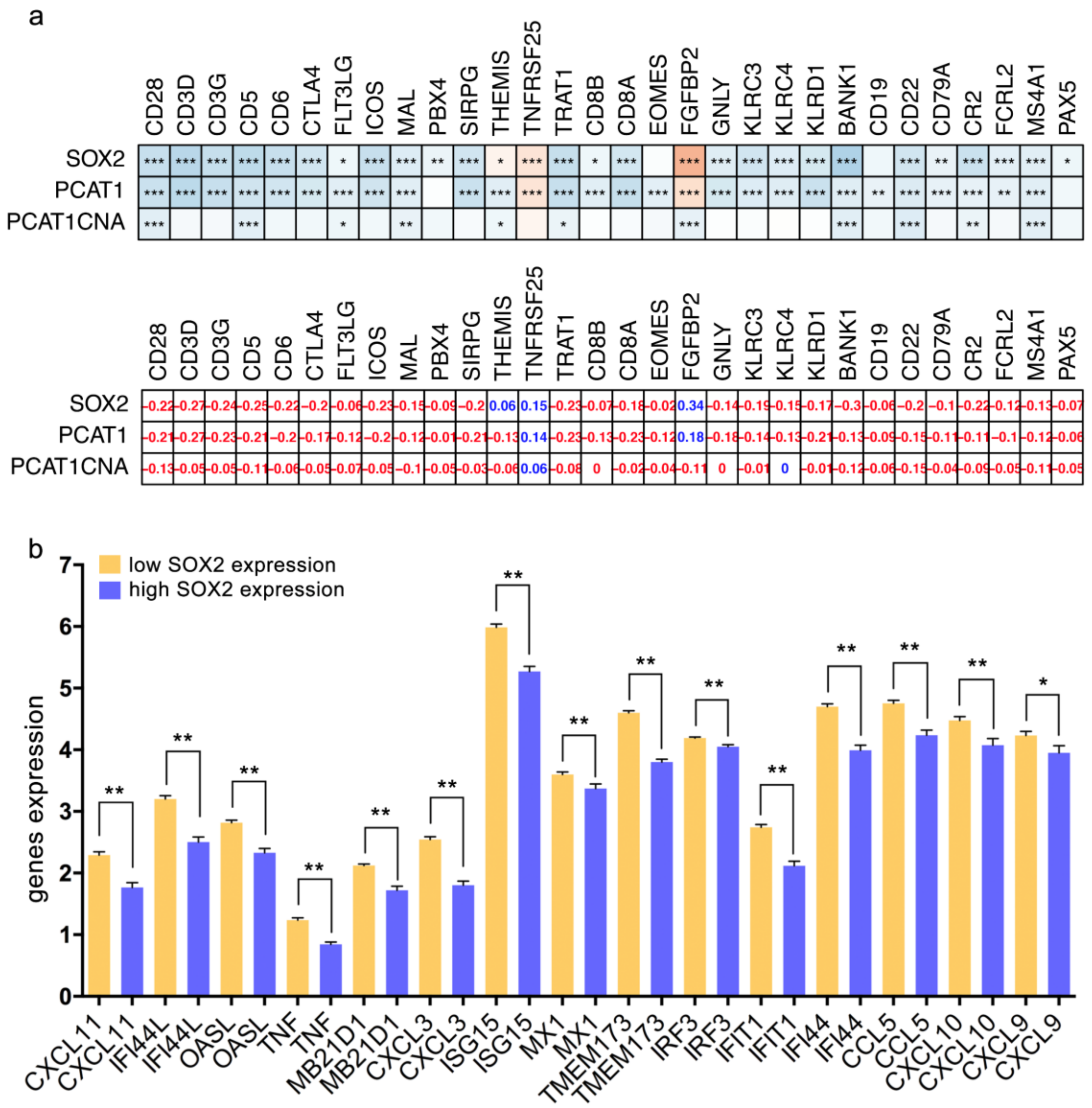

Figure S9. SOX2 potentiated an immunosuppressive tumor microenvironment. (a) MCPcounter was implemented to reveal SOX2 and PCAT1 expression. PCAT1 CNA was negatively correlated with major cellular markers of T cells, cytotoxic lymphocytes and B cells. (B) Heat map of representative gene expression between SOX2 low- and high-expression groups. Plotted Z-score of cGAS/STING pathway related genes and ISGs.

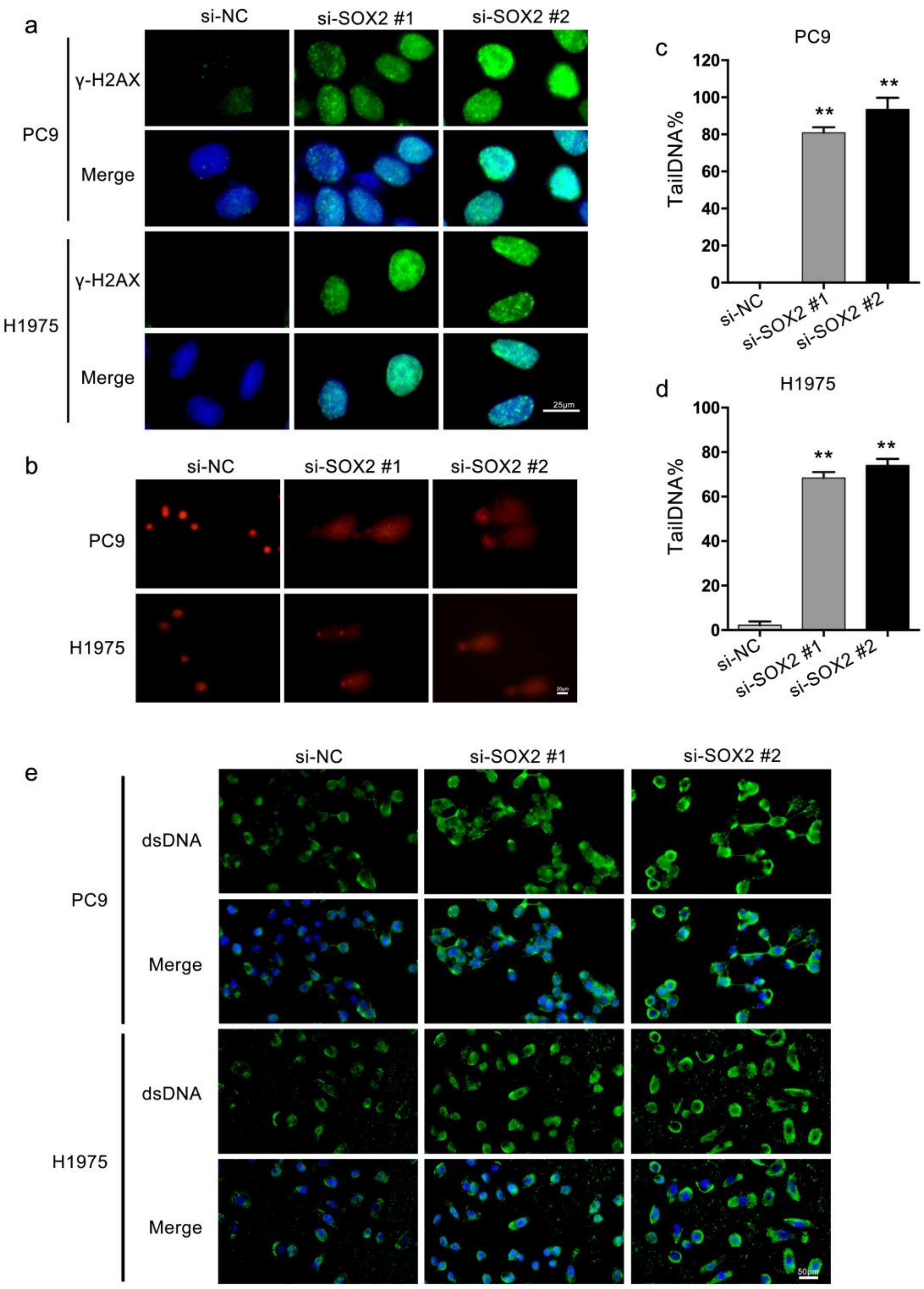

Figure S10. SOX2 silencing induced DNA damage. (a) Representative immunofluorescence of γH2AX. Scale bar: 25 μm. (b) Representative Comet assay in SOX2-deficient PC9 and H1975 cells. Scale bar: 20 μm. (c, d) The percentage of tail DNA content was quantified. (e) Representative immunofluorescence of dsDNA in SOX2-deficient PC9 and H1975 cells. Scale bar, 50μm. N = 3; **, $P < 0.01$.

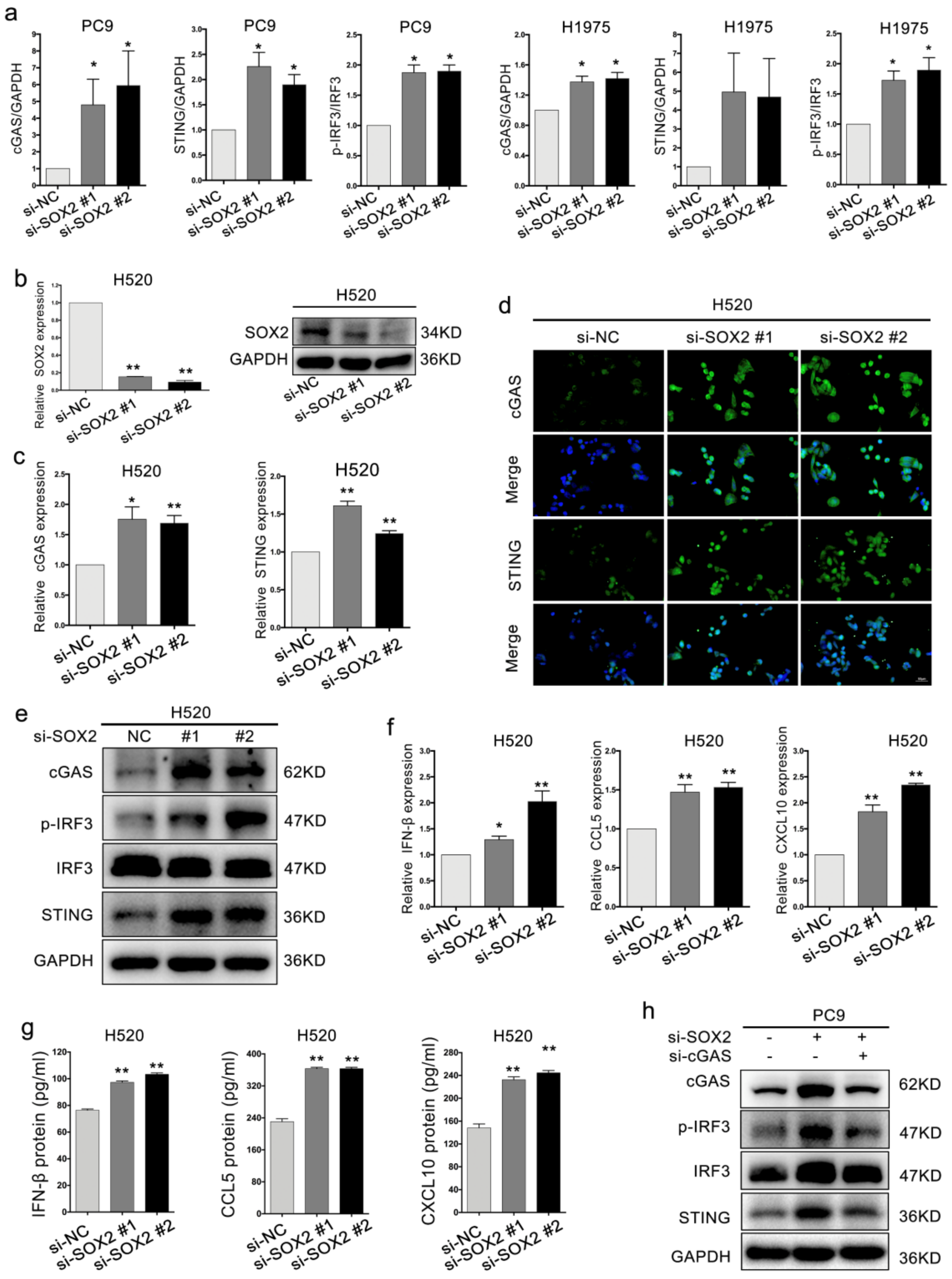

Figure S11. SOX2 regulated cGAS/STING signaling pathway. (a) Quantification for immunoblotting of cGAS/STING pathway proteins in SOX2-deficient PC9 and H1975 cells. (b) SOX2 was downregulated by siRNAs in H520 cells. (c) SOX2 knockdown increased cGAS mRNA levels in H520 cells. (d) Representative

immunofluorescence of cGAS and STING in SOX2-defecient H520 cells. (e) SOX2 depletion induced cGAS and STING protein levels, as well as IRF3 phosphorylation. (f) SOX2 downregulation increased IFN-β, CCL5 and CXCL10 mRNA levels in H520 cells. (g) More IFN-β, CCL5 and CXCL10 were secreted by the SOX2-deficient H520 cells. (h) cGAS depletion impaired the induction of cGAS/STING signaling pathway by SOX2 downregulation. N = 3; *, P < 0.05; **, P < 0.01.

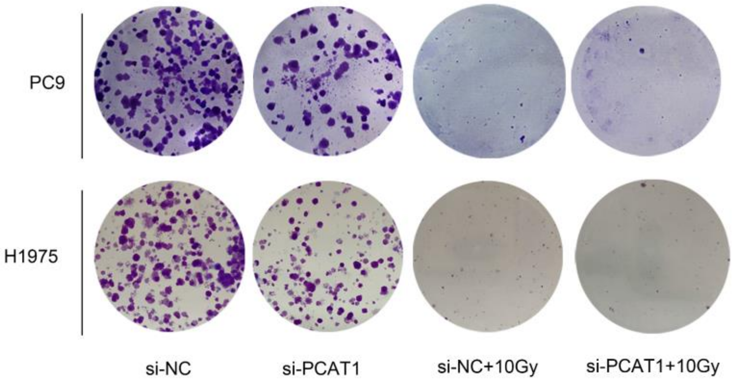

Figure S12. Colony formation assay of NSCLC cells with radiation at 10 Gy. IR at 10 Gy was too strong for both PC9 and H1975 cells at low density for this experiment.

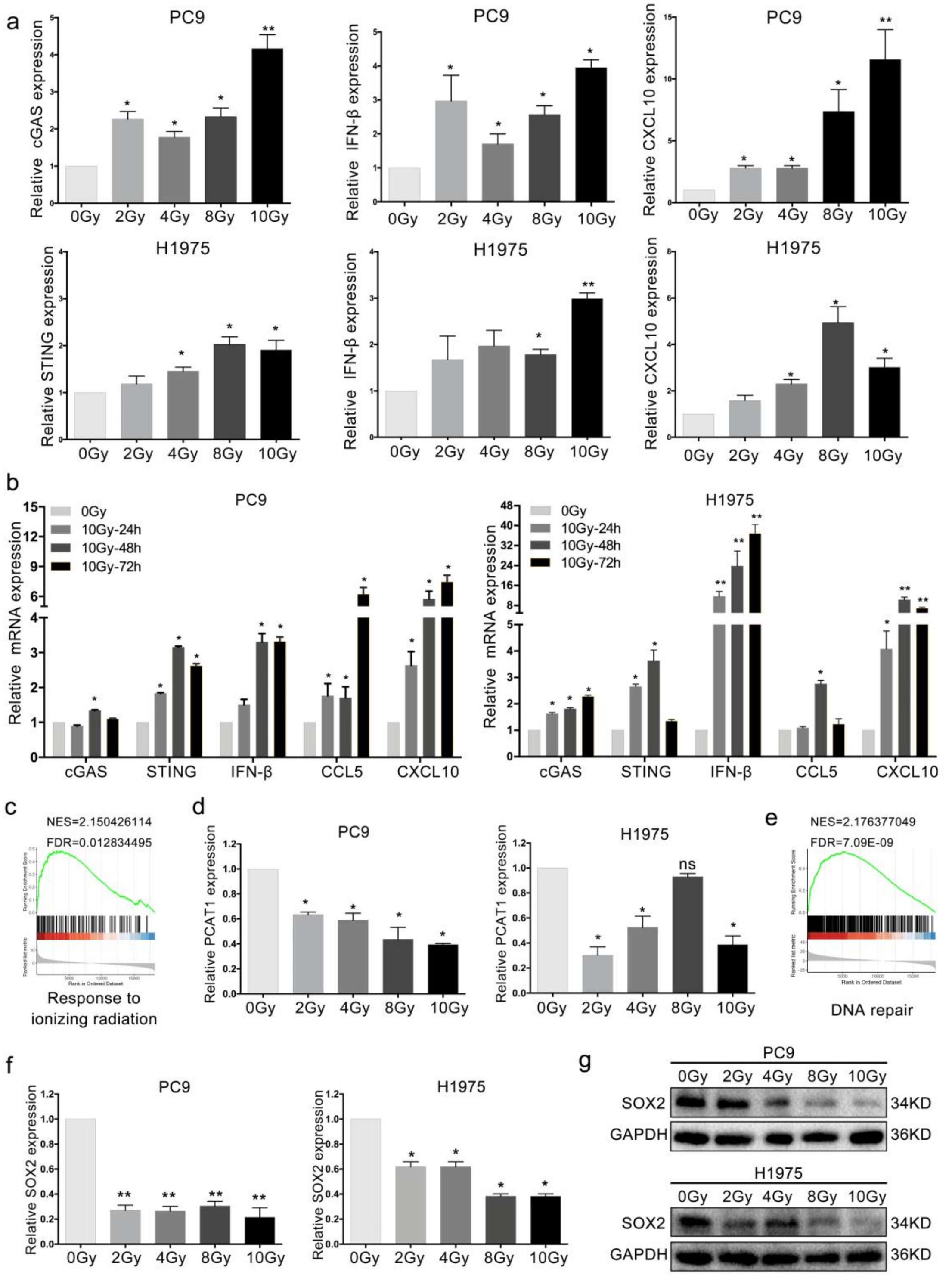

Figure S13. Radiation activated cGAS/STING signaling pathway and downregulated PCAT1 and SOX2 in NSCLC cells. (a) The mRNA levels of cGAS were detected in NSCLC cells treated with increasing doses of radiation (0, 2, 4, 8 and 10 Gy). (b) The mRNA levels of cGAS, STING, IFN-β, CCL5 and CXCL10 were detected 24, 48 and

72 h after radiation (10 Gy). (c) GSEA for the enriched gene sets in the PCAT1 expression with response to ionizing radiation. (d) The mRNA levels of PCAT1 were decreased in a dose-dependent manner in PC9 and H1975 cells. (e) GSEA for the enriched gene sets in the SOX2 expression associating DNA damage repair. (f) The mRNA levels of SOX2 were decreased in a dose-dependent manner in PC9 and H1975 cells. (g) The protein levels of SOX2 were diminished in a dose-dependent manner in PC9 and H1975 cells. N = 3; *, $P < 0.05$; **, $P < 0.01$.

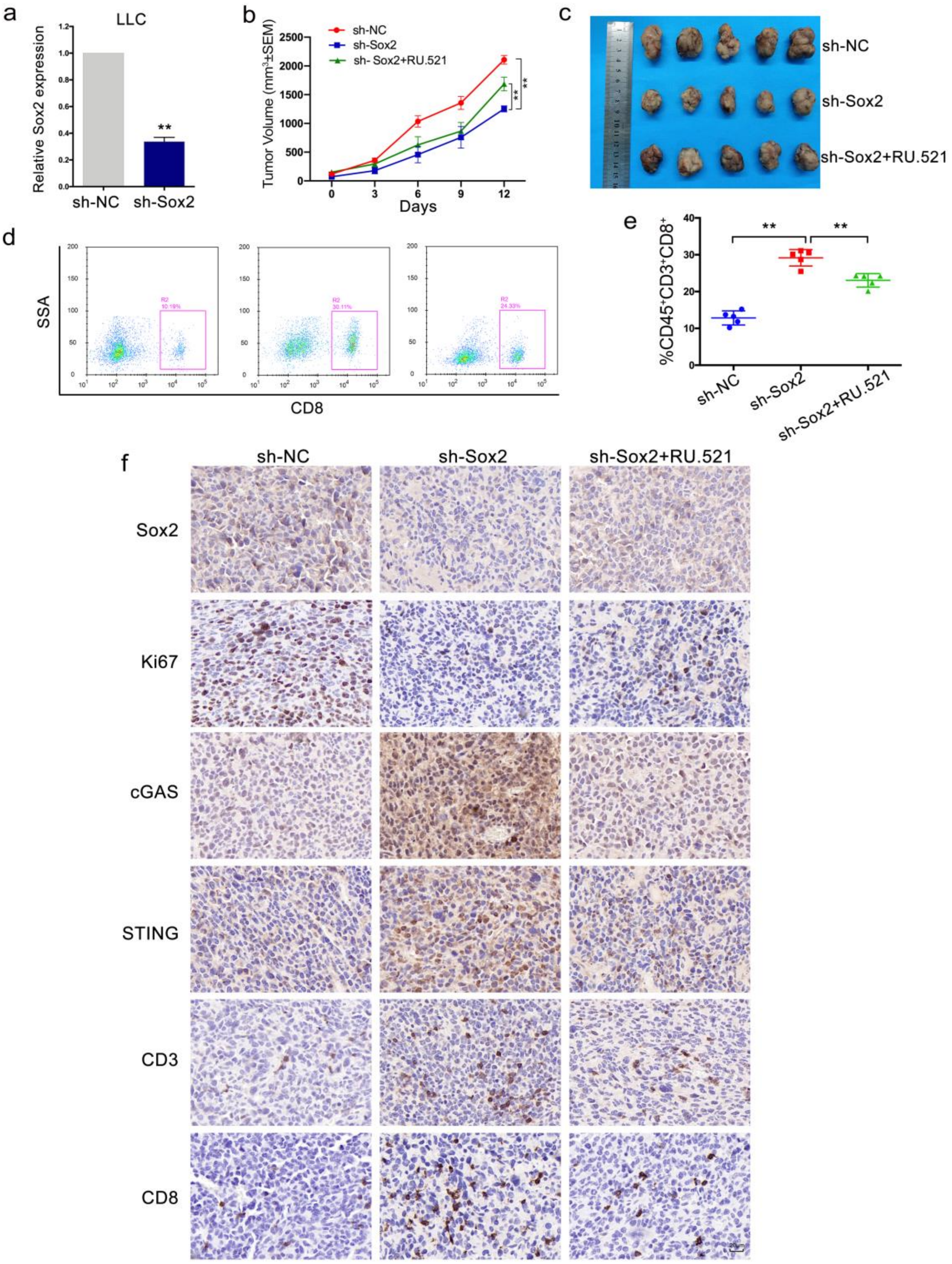

Figure S14. SOX2 silencing suppressed LLC cell growth via inducing CD8+ T cells through cGAS/STING pathway *in vivo*. (a) The efficiency of LV-sh-SOX2 was validated in LLC cells. (b) The tumor volumes were measured and depicted in the line chart. (c) Representative photos of tumor xenografts. (d) Representative flow cytometry of CD8+ T cells in the tumor tissues. (e) Quantification of CD8+ T cells in

mice with LV-sh-SOX2 LLC and RU.521 treatments. (f) Representative IHC images of SOX2, Ki67, cGAS, STING, CD3 and CD8 in tumor tissues. Scale bar, 20 μm.

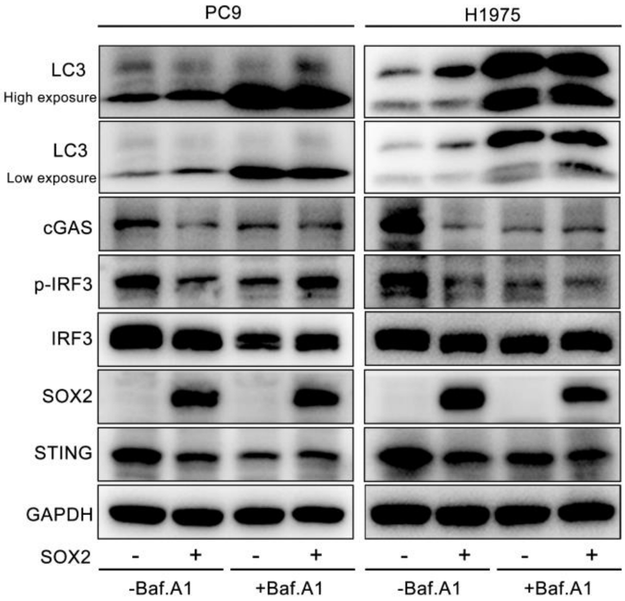

Figure S15. SOX downregulated cGAS/STING and downstream genes in an autophagy-dependent manner. PC9 and H1975 cells were transfected with SOX2-expressing plasmids and treated with bafilomycin A1 (200 nM) for 8h. Immunoblotting results indicated that autophagy inhibition partially restores LC3-II, cGAS and STING levels, suggesting that autophagy was involved in SOX2-mediated regulation of cGAS /STING pathway.
